# A low-complexity M-shaped reconfigurable intelligent meta-surface for mitigating pathloss in wireless systems

**DOI:** 10.1038/s41598-025-09741-1

**Published:** 2025-07-08

**Authors:** Maira Khafagy, Sherief Fathi, Ahmed Magdy

**Affiliations:** 1 Department of Physics, School of Science and Engineering, The American University in Cairo, New Cairo, Egypt; 2 Electrical Engineering Department, Suez Canal University, Ismailia, Egypt

**Keywords:** Path loss, Meta-surfaces, mm-wave, 6G, Indoor application, Engineering, Electrical and electronic engineering

## Abstract

Future 6G wireless communication systems require innovative solutions to overcome severe path loss, particularly in the millimeter-wave (mmWave) frequency bands. This study presents a novel Low-Complexity M-shaped Reconfigurable Intelligent Metasurface (LCM-RIM) designed to mitigate path loss in indoor environments. The proposed LCM-RIM features a compact, single-layer unit cell based on a low-loss Rogers substrate, offering a lightweight and cost-effective design suitable for seamless integration into wall-mounted installations in office and conference room settings. Each unit cell incorporates an AlGaAs PIN diode, enabling control at high frequencies and facilitating 1-bit phase modulation with discrete phase shifts of $0^\circ$ and $180^\circ$, operating at 24.12 GHz. This configuration supports passive beamforming with low hardware complexity and minimal power consumption. A $32 \times 32$ array configuration (1024 elements) with $0.5\lambda$ element spacing is used to enhance the gain. The LCM-RIM is employed to enable effective wavefront manipulation and ensure scalability for large-area coverage. To evaluate system-level performance, a numerical path loss model is developed by characterizing the angular gain profile of the LCM-RIM, which follows a Gaussian distribution across reflection angles. The model is validated using MATLAB simulations under various transmitter-receiver distances and angles of incidence. Results indicate that the LCM-RIM structure can enhance received signal strength by up to 15 dB in typical mmWave indoor scenarios. These findings underscore the potential of the proposed LCM-RIM design for practical deployment in future 6G networks, offering an efficient and scalable solution to address mmWave path loss in enclosed environments.

## Introduction

The world is progressing toward the sixth generation (6G) network, a transformative advancement aimed at making the world smarter. By improving the Quality of Service (QoS) and enabling intelligent environments such as smart cities, autonomous industries, and smart homes, 6G networks promise to redefine connectivity^1^. This evolution is driven by the integration of several technologies, including Light Fidelity (Li-Fi), Visible Light Communication (VLC), and Terahertz (THz) communications, which collectively pave the way for next-generation wireless systems^2,3^.

Over the past two decades, advances in physics have led to the development of artificial materials with unique properties not found in nature, such as photonic crystals^4^, meta-materials^5^, and meta-surfaces^6^. These materials are designed to control Electromagnetic (EM) waves in complex and scattering environments^7,8^. Meta-surfaces, which are the two-dimensional counterparts of meta-materials, are arranged in planar forms composed of specially configured meta-atoms. This structure offers cost-effective fabrication, lower energy consumption, and ease of implementation. By designing meta-atoms with specific characteristics, meta-surfaces can manipulate the propagation and scattering of EM waves, enabling physical effects such as wave reflection^9^, refraction^10^, and even the generation of detailed holographic images^11,12^. These capabilities make them suitable for emerging devices and applications, including ultra-thin meta-lenses for beamforming^13–15^ and meta-surface antennas^16^. With the advancement of passive meta-surfaces, limitations have emerged in certain applications due to their fixed functionality once fabricated. These surfaces are typically constrained by design, making them incapable of dynamic adjustments. Techniques based on polarization^17^, frequency^18–20^, or direction^21,22^ remain static and cannot be modified to offer real-time control.

To overcome this limitation, the concept of Reconfigurable Meta-surfaces (RM) was introduced. RMs incorporate external tuning elements into their resonant structures, enabling dynamic adjustment of reflection properties at specific phases in response to incident waves. These structures often include programmable circuits that allow control of EM waves across a broad frequency spectrum, including microwaves^23^, THz waves^24^, and optical regions^25^. Recent research has explored tunable components such as diodes^26–29^, graphene^30^, Liquid Crystals (LC)^31,32^, and Phase-Change Materials (PCM)^33^. Despite their advantages, RMs face design challenges—particularly achieving tunable performance within specific frequency ranges while managing complex biasing networks. These complexities hinder their implementation in high-frequency and large-scale applications.

**Fig. 1:**
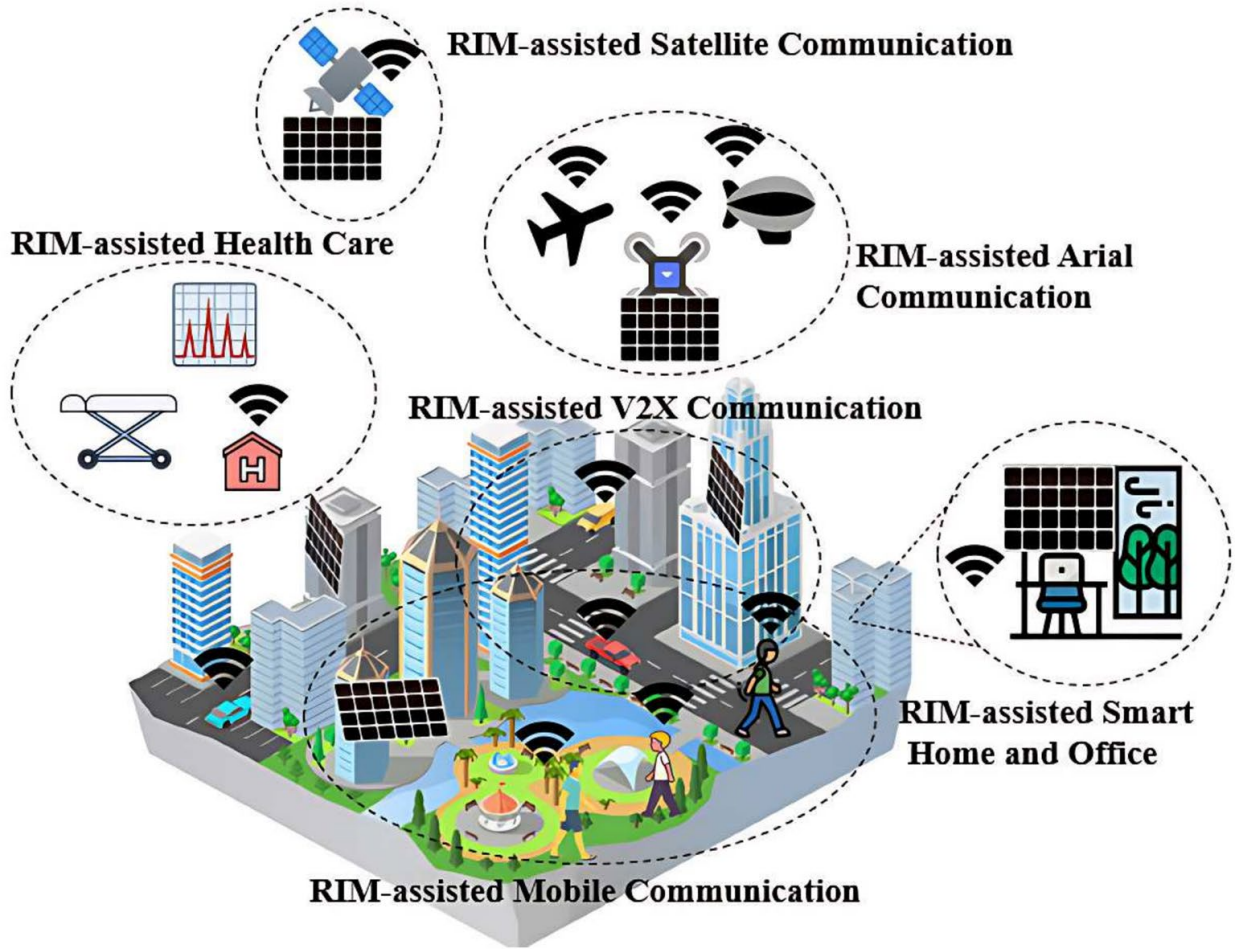
Reconfigurable intelligent meta-surfaces assistant smart applications.

To address these issues, Reconfigurable Intelligent Surfaces (RIS), also known as spatially-aware meta-surfaces, Reconfigurable Intelligent Meta-surfaces (RIM), or Intelligent Reflecting Meta-surfaces (IRM), have been proposed as a spatial extension of RM, offering a smarter and more adaptive way to manipulate EM waves^34,35^. RIMs resemble RMs in that both can control reflection or transmission to achieve tunable phase shifts^36^. However, RIMs go further by incorporating self-adaptive algorithms that allow intelligent control of incident EM waves with minimal or no human intervention^37^. This capability enables functions such as smart beamforming^38^, beam focusing^39^, and adaptive retroreflection^40^. This intelligence transforms RIM/IRM systems from passive reflectors into dynamic elements that support advanced applications, including RIM-assisted smart healthcare^41^, smart homes^42,43^, security^44,45^, smart cities^46^, the Internet of Things (IoT)^47^, and Vehicle-to-Everything (V2X) systems^48^, as illustrated in Fig. 1. These meta-surfaces can autonomously reconfigure beam paths to maintain ultra-reliable links, even in the presence of user mobility or environmental obstacles. While previous designs, such as those presented in^43,46^, have significantly advanced communication systems by incorporating RIS/RIM concepts, our work focuses specifically on the design of a novel RIM structure that introduces an M-shaped unit cell. This design offers several main advantages not simultaneously addressed in prior works, including enhanced phase tunability, broader angular stability, and a more compact design. These advancements collectively create a more efficient and adaptable reconfigurable metasurface, making it particularly suitable for use in dynamic indoor environments where effective beam control is essential.

### Related work

One of the primary characteristics of RIM is their ability to improve coverage in complex environments, such as urban areas where the Line of Sight (LoS) is often obstructed. Path loss model predictions enable RIM to automatically adjust its amplitude and phase, making the development of accurate path loss models essential for optimizing RIM configuration and conducting link budget analysis. In both theoretical and experimental research, such as the work presented in^48^, a free-space path loss model for RIS-assisted wireless communications has been developed. This model incorporates physical characteristics of the RIS, including the unit cell size and radiation patterns, while distinguishing between near-field and far-field propagation scenarios. By addressing challenges related to beamforming and broadcasting applications, the authors validated their approach through simulations and experimental measurements. The study in^49^ proposes a generalized free-space path loss model for IRS-assisted THz Multi-Input Multi-Output (MIMO) wireless systems. Their analytical framework derives closed-form expressions for various scenarios, including general cases, far-field and near-field conditions, phase-induced beamforming (PiBF), and phase-induced broadcasting (PiBS), by accounting for factors such as antenna radiation patterns, IRS configuration, and atmospheric molecular absorption. The results show that, under far-field conditions, configuring the IRS based on the angles of arrival and departure can significantly reduce path loss. In^50^, a method-of-moments-based approach is used to analyze frequency-dependent path loss characteristics in IRS-assisted Single-Input Single-Output (SISO) links. The study indicates that, in addition to the IRS aperture, the dimensions and resonant modes of individual IRS unit cells significantly influence the wideband path loss behavior. This results in ripple-like variations, or scalloping, of approximately 4 to 6 dB, caused by the appearance of additional side lobes in the radiated beam. The work in^51^ presents a spatial scattering channel model-based path loss framework for cooperative double-IRS-aided systems. Their model quantifies the cascaded path loss of the double reflection link by incorporating main factors such as the physical size and gain of IRS elements, along with near- and far-field propagation effects between the IRSs. Notably, the authors derive a closed-form expression demonstrating how inter-IRS collaboration and optimal element allocation (e.g., equal distribution between the two IRSs) can maximize received signal power. In^52^, a path loss model tailored for RIS-assisted wireless communication in tunnel scenarios is developed. By calibrating their simulation platform with field measurements from Shanghai Metro Line 7, the authors extend traditional free-space models using ray tracing and EM theory to capture the unique propagation characteristics of tunnel environments. Their model incorporates a t-distribution-based correction factor to address the inherent uncertainty in such scenarios and shows that RIS deployment can reduce path loss by approximately 20 dB, significantly enhancing signal transmission in rail transit systems.

While recent studies have focused on RIS applications in outdoor wireless communications—including spatial deployment, channel modeling, and path loss reduction in urban and tunnel environments—this paper diverges by concentrating on indoor scenarios. Specifically, we investigate how RIM can mitigate the adverse effects of path loss in enclosed spaces, where challenges such as multipath fading and limited coverage are prevalent, by developing an indoor-specific path loss model.

### Motivation and contributions

This work aims to enhance future 6G wireless networks by developing a novel LCM-RIM based on AlGaAs PIN diodes as hardware simplicity and scalability for passive indoor mmWave enhancement. The key contributions of this research are summarized as follows:

- **Design and simulation:** This work introduces a low-complexity meta-surface (LCM-RIM) featuring a novel “M”-shaped, single-layer unit cell operating at 24.12 GHz, optimized for mmWave indoor applications. The structure is fabricated using conventional ROGERS materials to ensure low loss at high frequencies. The lightweight and compact design facilitates easy deployment on walls or fixed indoor surfaces, targeting improved coverage in enclosed environments such as meeting rooms and conference spaces.
- **1-bit phase control using AlGaAs PIN diodes:** Each LCM-RIM unit cell integrates a single AlGaAs PIN diode to achieve binary (1-bit) phase modulation with discrete $0^\circ$ and $180^\circ$ phase shifts. These diodes are chosen for their low power consumption and suitability for high-frequency switching, enabling passive beamforming with minimal hardware complexity.
- **Scalable lightweight LCM-RIM:** To demonstrate practical feasibility and scalability, the LCM-RIM is expanded to large array sizes (e.g., 32$\times$32), offering 1024 discrete control elements. The array maintains a compact inter-element spacing of 0.5$\lambda$, ensuring high efficiency in beam redirection and signal focusing, even in large indoor scenarios.
- **Numerical path loss model:** A numerical path loss model is developed based on the LCM-RIM gain profile and effective aperture. The gain variation with incident angle is characterized using a Gaussian function, fitted from unit cell behavior under different angles. This model captures the directional reflection performance of the meta-surface and provides accurate received power predictions under practical conditions.
- **Validation of the LCM-RIM path loss effectiveness:** The effectiveness of the LCM-RIM is validated using MATLAB-based system-level simulations. The results confirm significant improvements in received power under varying incident angles and Rx–RIM distances. Evaluations using different array sizes (16$\times$16, 32$\times$32, 64$\times$64) show that the proposed LCM-RIM can enhance mmWave link performance by up to 15dB, supporting its scalability and practical utility in real-world scenarios.

**Fig. 2:**
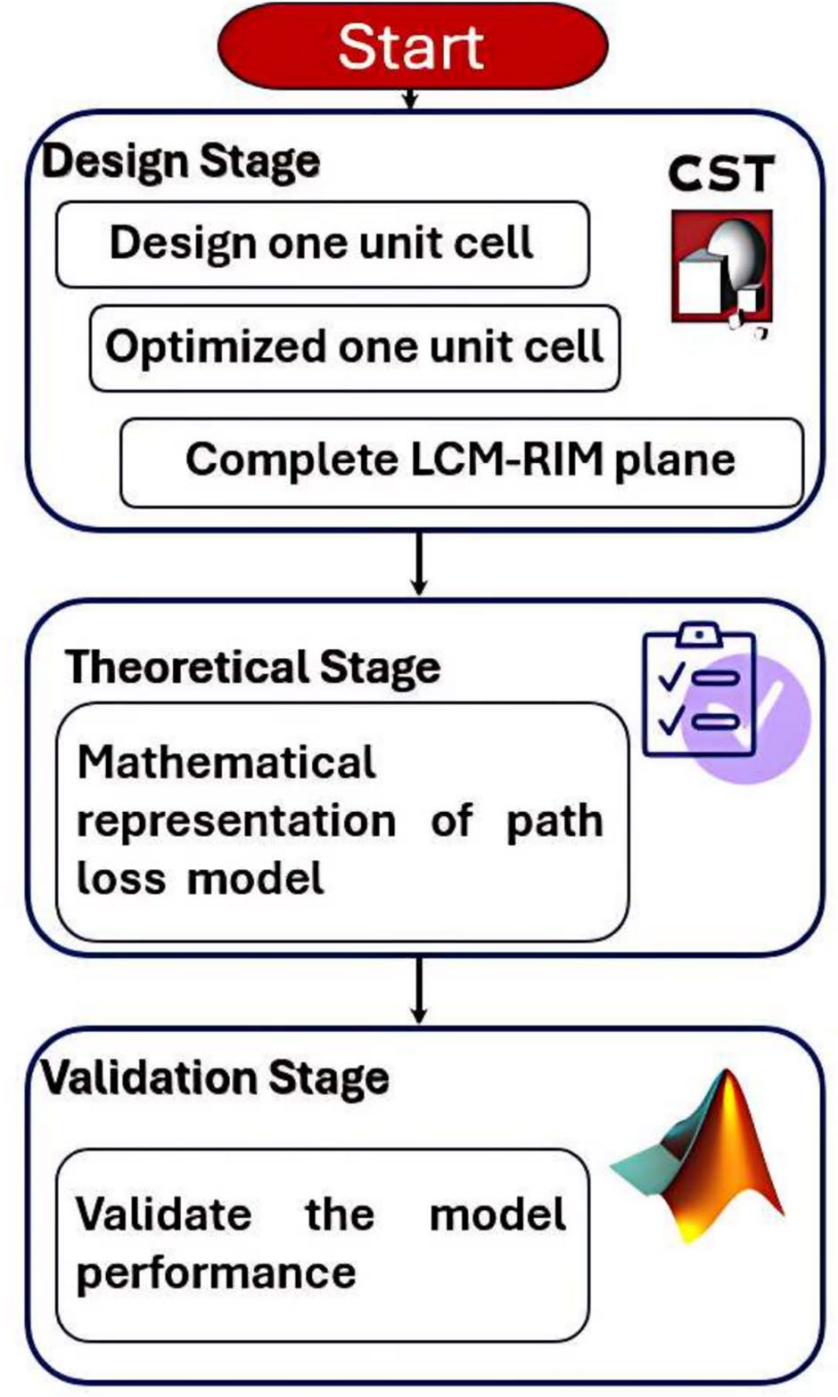
Flowchart of the development process comprising design, theoretical analysis, and simulation validation.

This paper presents a comprehensive approach to the design, modeling, and validation of the proposed LCM-RIM for indoor mmWave applications. As illustrated in Fig. 2, the workflow consists of three main stages. In the design phase, the individual unit cell is first developed and optimized, followed by the construction of the complete LCM-RIM array using CST Microwave Studio. In the theoretical phase, a mathematical path loss model is formulated to capture the behavior of mmWave signal propagation in the presence of the meta-surface. Finally, in the validation phase, the effectiveness of the proposed model is assessed using MATLAB simulations under various operating conditions. This structured approach ensures that the proposed LCM-RIM design effectively bridges the gap between EM design and system-level performance modeling.

This paper is organized as follows: section “Introduction” introduces the system model of meta-surfaces. Section “Related work” presents the novel design of the LCM-RIM in the CST simulator. Section  “System model” describes the proposed path loss model. Section “Proposed design of LCM-RIM” validates the design for indoor applications using $16 \times 16$, $32 \times 32$, and $64 \times 64$ arrays. Section “Path loss modeling for proposed LCM-RIM” discusses the measurement setup used to evaluate the received power in the lab. Finally, the study is summarized in the “Conclusion”.

## System model

This section discusses the principles of RIM, with a focus on its structure and control mechanisms for adaptive beamforming. These foundational concepts will be important in the following subsections, where the design and path loss model are presented in detail. First, we introduce the design and functionality of the RIM in 1.1. Then in 1.2, we discuss how beamforming and passive gain of the RIM can enhance performance.

### Design and functionality of RIM

**Fig. 3:**
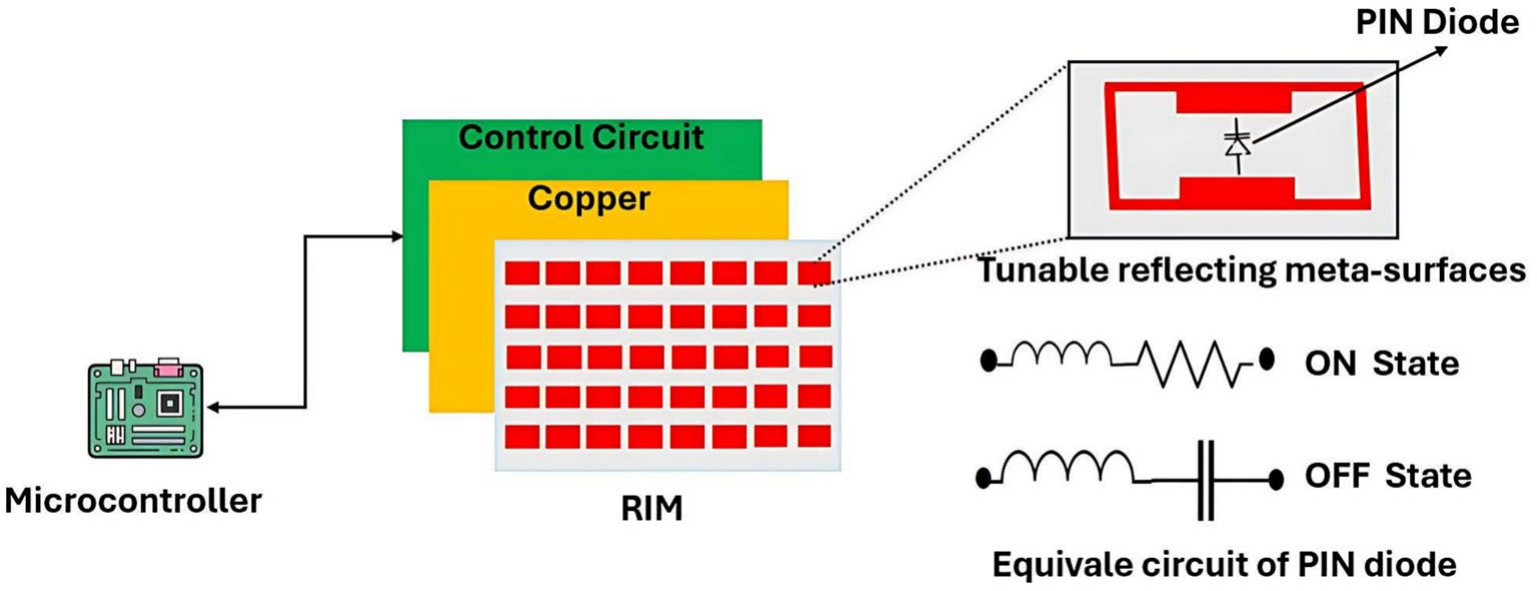
Structure of the passive RIM.

The passive programmable RIM consists of a two-dimensional array of reflecting elements, such as meta-atoms, designed to control the EM wave^53,54^. These elements can be designed with geometrical properties, such as shape, size, and arrangement, to achieve the desired signal response. The RIM is designed to adjust the reflection properties (amplitude and phase), which makes it more intelligent to adapt the propagation of the channel^55^. Figure 3 illustrates the general structure of the RIM, which is composed of three layers. The first layer is the resonator layer, consisting of meta-surface patches mounted on a dielectric substrate. These resonators can be fabricated from tunable materials such as LC^56^ or vanadium dioxide ($VO_2$)^57^, or they can employ external active tuning elements such as PIN diodes^58^ or varactor diodes^59^. These active elements provide control over the reflection characteristics of the resonator elements. In this study, we focus on the use of PIN diodes as tuning elements, which can effectively control the phase of reflected waves in highly attenuated environments. The second layer is a copper backing layer, which acts as a ground plane to prevent signal leakage. The third component is the external control circuit, which adjusts the phase and reflection coefficients to achieve the desired beamforming^60,61^. The control unit is typically implemented using a Microcontroller Unit (MCU) or a Field-Programmable Gate Array (FPGA), enabling physical coding to optimize phase shifts based on feedback from the Channel State Information (CSI).

### Beamforming and passive gain

Building on the previous subsection, we now discuss the beamforming characteristics of the passive RIM. This includes the concept of passive gain and the corresponding electric field equations, which are essential for understanding the proposed path loss model discussed in the following sections.

**Fig. 4:**
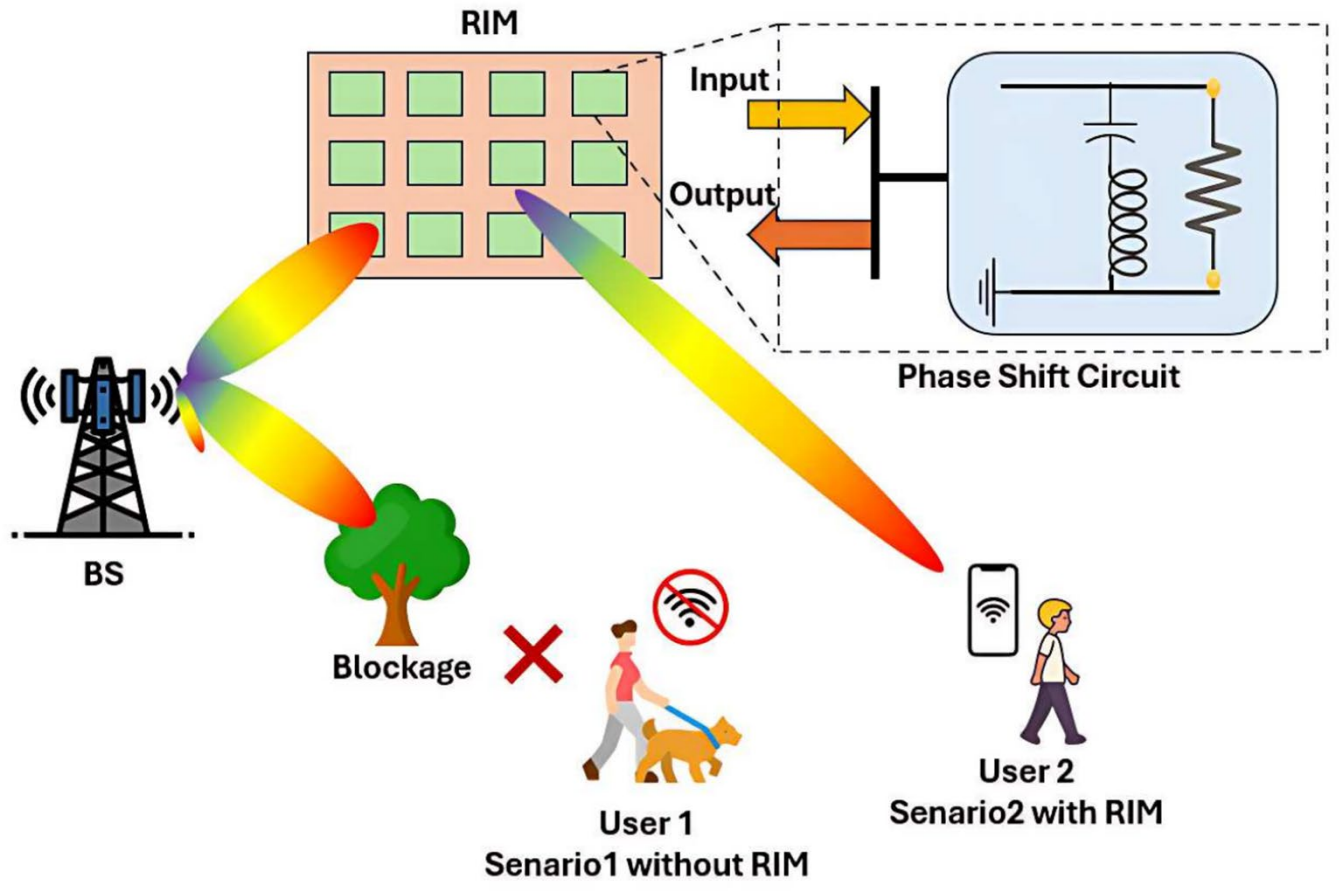
Illustration of a RIM redirecting signals around a blockage to maintain wireless connectivity for the user.

Beam steering in RIM can be achieved through binary phase control, as illustrated in Fig. 4. In this scenario, the RIM dynamically tracks user movement and reflects EM waves by adjusting the phase-shift circuit at the appropriate unit cell. Each unit cell selects a phase shift of either 0 or $\pi$, depending on the state of the PIN diode. The resulting beam steering can be mathematically described by considering the phase difference between the incident and reflected waves at each unit cell^62^.

1 $$\begin{aligned} \phi _{xy} = \phi _{r}(x_x, y_y) - \phi _{i}(x_x, y_y), \end{aligned}$$

where, *x* and *y* represent the positions in the RIM, $\phi _{xy}$ represents the resulting phase at *x* and *y* of the unit cell, $\phi _{i}(x_x, y_y)$ is the phase of the incident wave and $\phi _{r}(x_x, y_y)$ is the phase of the reflected wave. Since the PIN diode can be switched between ON and OFF states, the phase $\phi _{xy}$ is set to 0 when

$$\begin{aligned} -\frac{\pi }{2} \le \phi _r(x_x, y_y) - \phi _i(x_x, y_y) \le \frac{\pi }{2}, \end{aligned}$$

and to $\pi$ otherwise.

The radiation pattern $E_{r}(\theta , \varphi )$ generated by the RIM can be written as^63^:

2 $$\begin{aligned} E_{r}(\theta , \varphi )&= \cos (\theta ) \sum _{x,y=1}^{N} \Gamma _{xy} E_{i}(x_x, y_y) \cos (\theta _{xy}) \times \exp \left( -j k \sin (\theta )[x_x \cos (\varphi ) + y_y \sin (\varphi )]\right) , \end{aligned}$$

where $\cos (\theta )$ represents the radiation pattern of a single unit cell, $\Gamma _{xy}$ is the reflection coefficient at *x* and *y*, $E_{i}(x_x, y_y)$ is the electric field, $\theta _{xy}$ is the incident angle at *x* and *y*, and *k* is the wavenumber.

The next section introduces the proposed LCM-RIM design. This new design builds on RIM, integrating innovative approaches that optimize the performance of 6G.

## Proposed design of LCM-RIM

This section introduces the novel design of the LCM-RIM to mitigate path loss in complex environments. Introducing the design process, from a single unit cell to a large-scale array on the CST Studio Frequency Domain Solver. The following subsections describe each phase of the design process.

### Initial design of LCM-RIM single unit cell

The design of a single unit cell in the LCM-RIM is based on the structure of a resonant element that controls incident EM waves. In our approach, the unit cell consists of a resonator element embedded in the patch, coupled with parasitic elements to enable reconfigurability. These parasitic elements are strategically positioned such that their equivalent impedance varies according to the state of the switching diode, resulting in a $0^\circ$, and $180^\circ$ phase shift between the ON and OFF states^60^. The simulation boundary conditions are set to periodic, ensuring that the unit cell behaves as part of an infinitely repeating structure. A plane wave is incident from the front to analyze the unit cell’s response to EM waves. Floquet ports are used to model the excitation and capture the propagation effects, allowing for the excitation of multiple diffraction orders and enabling a detailed evaluation of the unit cell’s scattering and reflection characteristics.

**Fig. 5:**
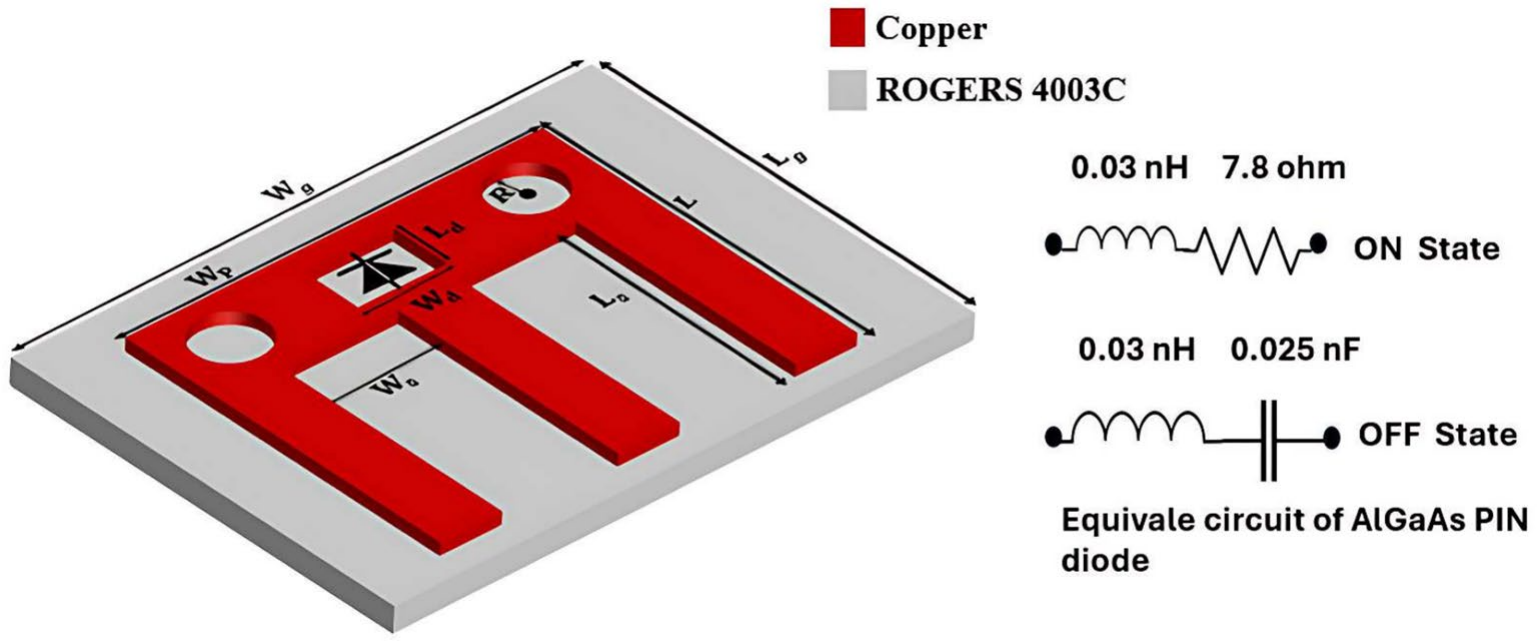
Perspective view to LCM-RIM.

Figure 5 illustrates the structure of the proposed LCM-RIM. The unit cell is constructed on a ROGERS 4003C substrate, which has a relative permittivity of 3.38 and a thickness of 0.2 mm. This material is selected for its low dielectric loss, making it suitable for high-frequency applications. In this work, an M-shaped structure is employed in the design of the LCM-RIM due to its effective control of EM waves. Unlike more complex RIM configurations, the unique single-layer M-shaped design simplifies fabrication, reduces cost, and maintains high efficiency. The proposed LCM-RIM also provides a highly stable phase shift that remains consistent even as the array size increases, a challenge often encountered in alternative RIM designs. Moreover, simulations show that this structure effectively mitigates path loss in complex environments. These advantages position the M-shaped RIM as a practical and high-performance solution for next-generation communication systems. A detailed analysis of its path loss mitigation capabilities will be presented in the following section.

The unit cell is designed to operate in two distinct states (ON and OFF), represented by phase shifts of 0 and $\pi$, respectively. A MADP-000907-14020P-AlGaAs PIN diode is employed to enable the tuning mechanism. This diode offers fast switching capabilities between the two states, which is crucial for maintaining control of EM waves in high-frequency ranges where signal attenuation is typically significant.

In the ON state, the PIN diode behaves as a series combination of inductance ($L_p$) and resistance ($R_p$). In the OFF state, it functions as a series combination of inductance ($L_p$) and capacitance ($C_p$), as illustrated in Fig. 5. The impedance of the PIN diode in both states is expressed as follows:

3 $$\begin{aligned} Z_{L_{\text {PIN}}}&= R_p + j\omega L_p \quad \text {(ON State)} \end{aligned}$$

4 $$\begin{aligned} Z_{L_{\text {PIN}}}&= j\omega L_p + \frac{1}{j\omega C_p} \quad \text {(OFF State)}, \end{aligned}$$

The reflection coefficient ($\Gamma _p$) is given by:

5 $$\begin{aligned} \Gamma _p = \frac{Z_{L_{\text {PIN}}} - Z_{R_p}}{Z_{L_{\text {PIN}}} + Z_{R_p}} e^{j\phi } \end{aligned}$$

where, $Z_R{_p}$ is the impedance of the unit cell. We design the unit cell to achieve an optimum phase of $180^o$ difference between the ON and OFF states at the center frequency of 24.12 GHz.

The fast-switching capability of the AlGaAs PIN diode enables the LCM-RIM to adjust its configuration in real time, making it suitable for dynamic wireless environments. This binary design of the proposed metamaterial results in lower power consumption and reduced system complexity compared to multi-bit RIM architectures. LCM-RIM is highly responsive to changes in its configuration (array pattern), enabling real-time optimization of the phase profile based on the Angle of Arrival (AoA) and Angle of Departure (AoD) of users. The LCM-RIM can direct beams toward specific receivers while generating nulls to reduce interference from unwanted signals. This spatial filtering capability allows the LCM-RIM to support Spatial Division Multiple Access (SDMA), allowing multiple users to operate within the same frequency band and mitigating interference.

In the next subsection, we will analyze the effect of the main parameter of the proposed M-shaped structure and define the main parameter that affects its behavior to achieve the desired reflection characteristics.

### Parameter optimization

**Fig. 6:**
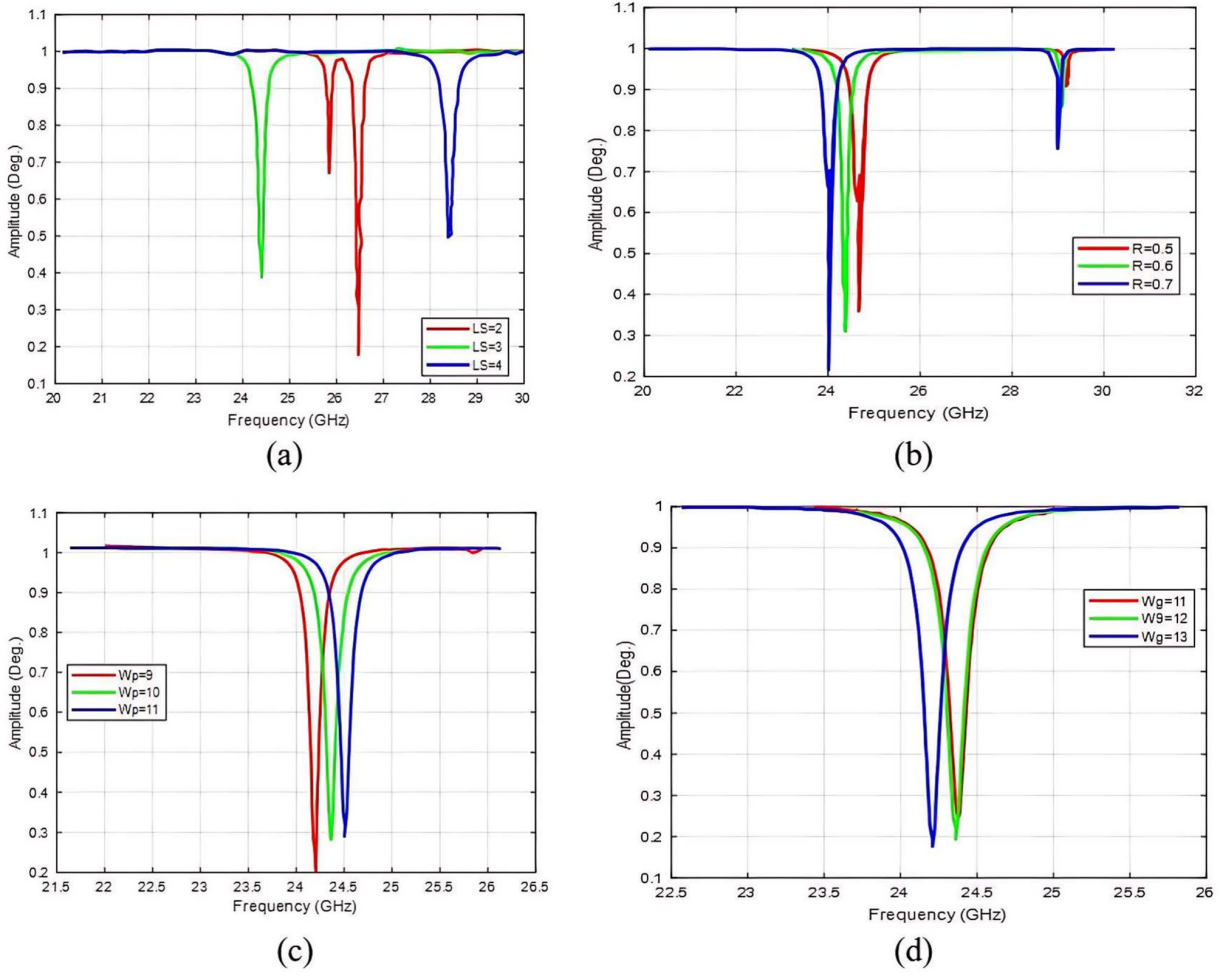
Parameter sweep of the proposed M-shaped design: (**a**) Ls, (**b**) R, (**c**) Wp, (**d**) Wg.

The effect of different parameters of the M-shaped structure is analyzed better to understand the behavior of the proposed LCM-RIM characteristics. Figure 6 shows the amplitude response as a frequency function for each M-shape’s main parameter, identified during the optimization stage. These parameters include the strip length ($L_s$), the radius (*R*), the patch width ($W_P$), and the ground width ($W_g$). To ensure the correct positioning of the PIN diode, the parameters $L_d$, (diode position length) and $W_d$ (diode position width) are set to 1 mm.

Figure 6a illustrates the variation in $L_s$ concerning the resonance frequencies, where a large $L_s$ shifts the resonance towards low frequencies. Figure 6b shows the effect of *R*, which shows that a small variation in *R* causes a slight shift in resonance and a change in amplitude. Figure 6c presents $W_p$, the changes in $W_p$ shift of the resonance frequency, highlighting its role as a tuning parameter. Lastly, Fig. 6d depicts the effect of $W_g$, which determines the unit cell size and influences the bandwidth and resonance. A larger variation in $W_g$ results in a slight shift in the resonance.

**Table 1:** Optimized parameters of M-shaped structure.

Parameter	Value
$L_s$	3 mm
*R*	0.6 mm
$W_d$	1 mm
$L_d$	1 mm
$W_s$	3 mm
$W_p$	10 mm
*L*	6 mm
$L_g$	10 mm
$W_g$	12 mm

**Fig. 7:**
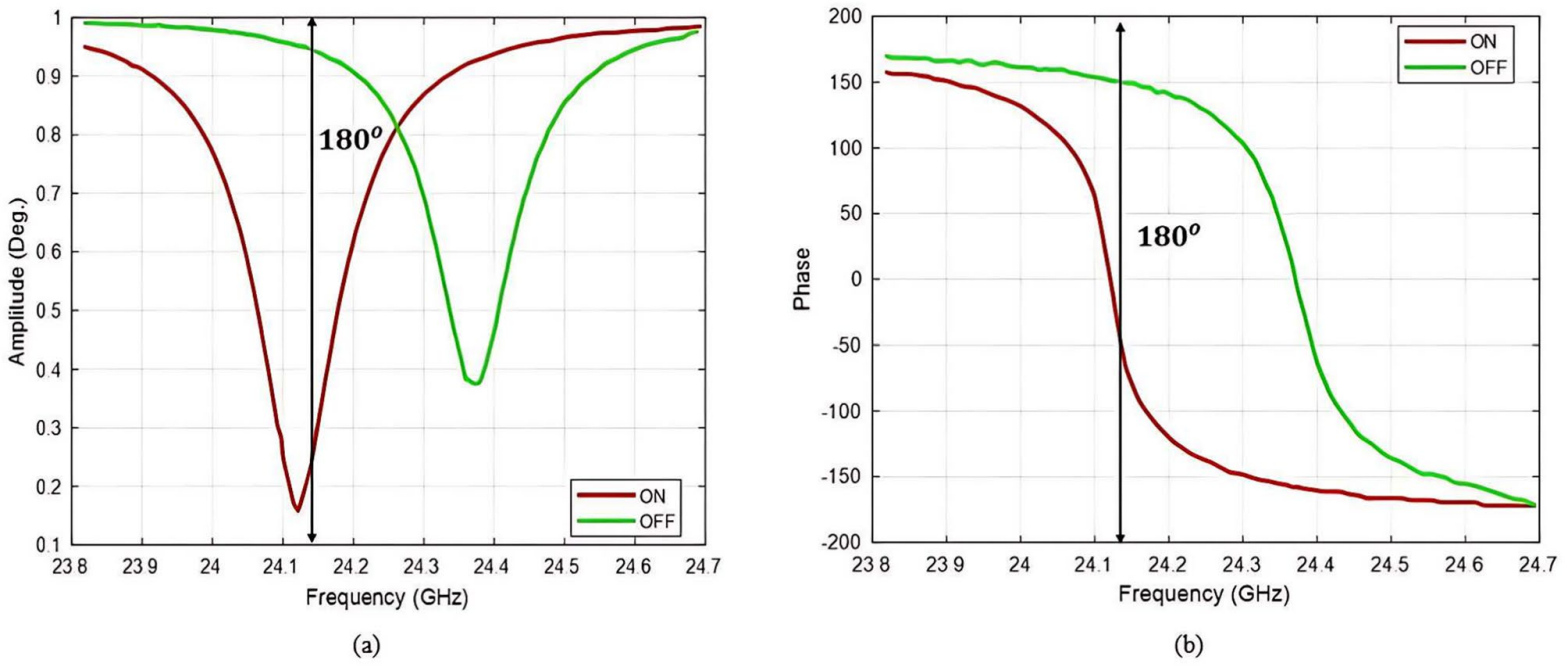
Reflection characteristics of the proposed LCM-RIM (**a**) amplitude and (**b**) phase.

Figure 7 represents the performance of the proposed single unit cell with 1-bit reconfigurable. The design has a single resonance at a peak of 24.12 GHz in the normal incident where ($\theta$ = 0, $\phi$ = 0). The resonance frequency remains constant in both the on and off- states except for the peak of magnitude. Figure 7a, represents the proposed LCM-RIM reflection amplitude response. The amplitude remains consistently high across the operating frequency range. Figure 7b illustrates the response of the reflection phase, achieving a $180^o$ phase shift. The AlGaAs PIN diode provides fast switching between the ON and OFF states. Table 1 provides a detailed list of the values of the optimized parameters for the M-shaped structure.

After the parameters are optimized, the design can be expanded into a multielement array, setting the stage for the next phase of the 2$\times$2 structure.

### Design 2x2 array of the proposed LCM-RIM

**Fig. 8:**
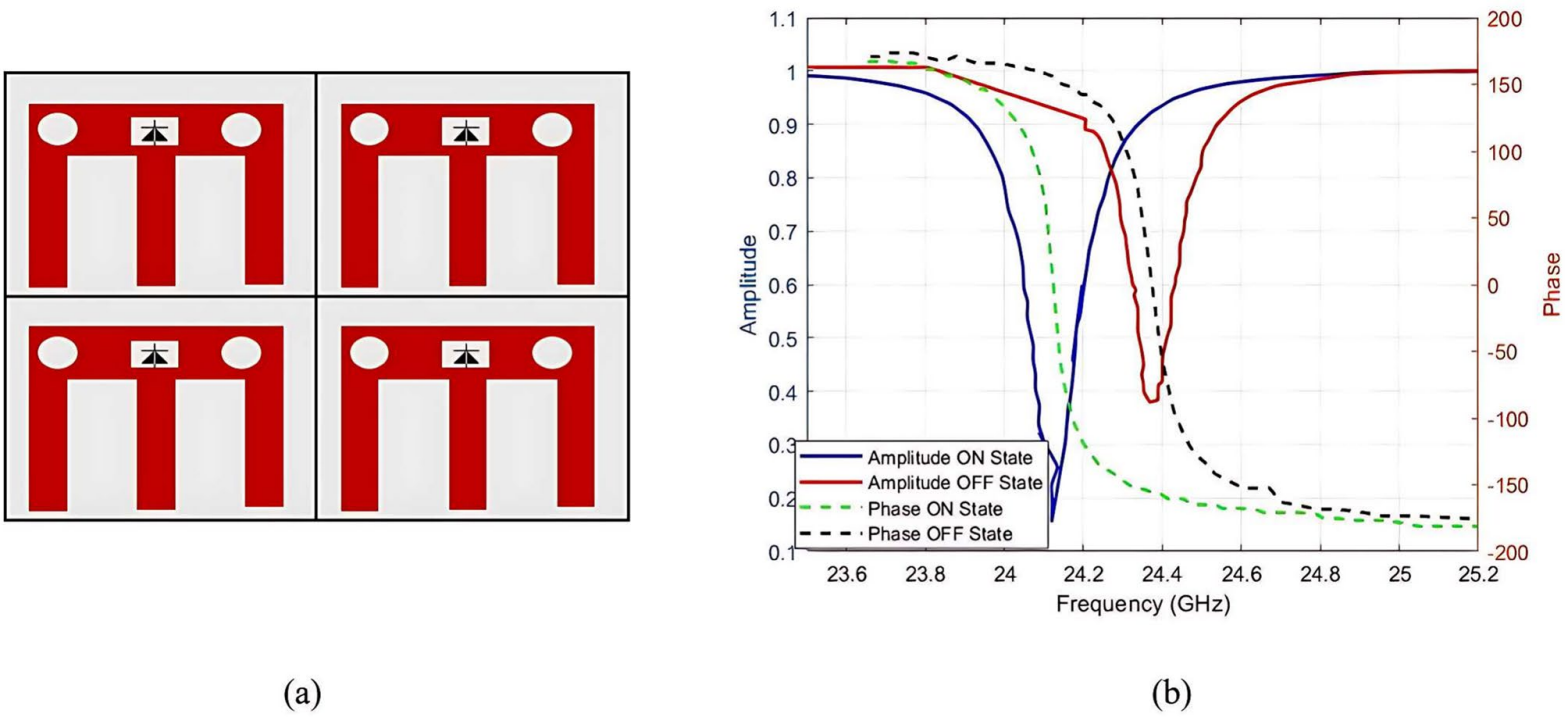
Proposed 2x2 LCM-RIM (**a**) schismatic diagram of 2x2, and (**b**) reflection characteristics amplitude and phase.

A critical stage in this study involves the development of a 2$\times$2 LCM-RIM structure to validate the fundamental principles governing the interaction of EM waves with the RIM. This configuration enhances control over EM wave propagation by increasing the number of tunable elements, thereby improving signal strength and enabling precise beamforming in multi-user environments across two spatial dimensions. Figure 8a presents the proposed 2$\times$2 array structure, which comprises four uniformly arranged, independently controllable unit cells. The horizontal spacing between passive elements is 0.5$\lambda$, while the vertical spacing is 0.4$\lambda$. This compact array serves as a preliminary platform for performance evaluation before scaling to larger configurations. As shown in Fig. 8b, the amplitude and phase responses remain stable in both operational states. The amplitude ripple is limited to 0.1, and the phase shift demonstrates a maximum deviation of 179$\circ$, indicating reliable and consistent performance across the array.

Although the proposed 2x2 LCM-RIM improves dynamic performance, small-scale arrays exhibit limitations in angular coverage for practical applications, which struggle to suppress dense multipath scattering in large-scale environments. To address these limitations, the proposed design was scaled to a 32$\times$32 architecture, increasing the number of elements from 4 to 1024. The next subsection discusses this large scaling process in detail.

### Design and analysis of a large-scale array of the proposed LCM-RIM

To overcome the limitations identified in the 2$\times$2 design, the proposed structure was scaled up to a 32$\times$32 array. This large-scale configuration significantly increases the number of elements, thereby enhancing the control performance of the LCM-RIM. The primary objective of this expansion is to improve spatial coverage in indoor environments, such as smart homes and high-priority areas like conference rooms.

**Fig. 9:**
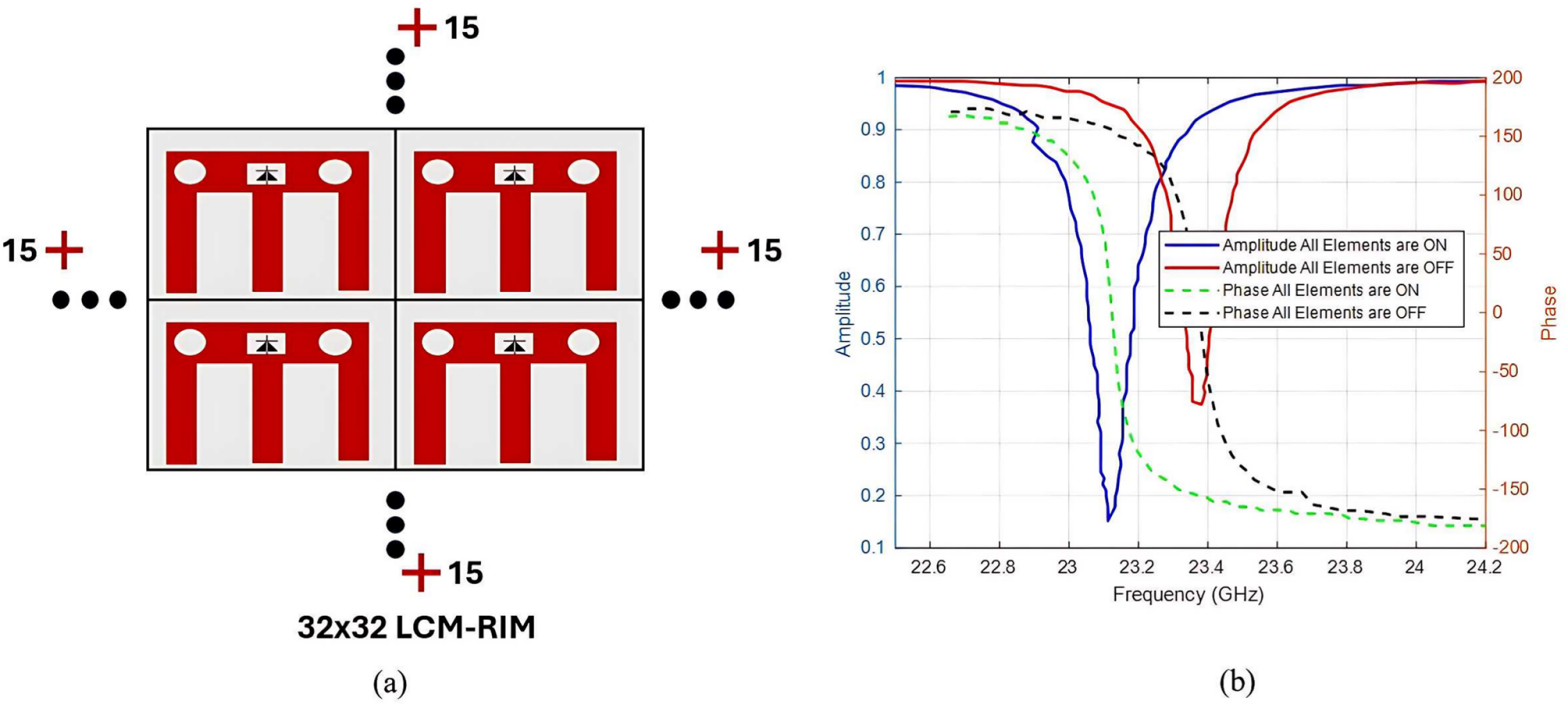
Proposed 32 LCM-RIM (**a**) schematic diagram 32$\times$32 array, (**b**) amplitude and phase response when all elements are ON and when all elements are OFF.

Figure 9a illustrates the proposed 32x32 LCM-RIM, Fig. 9b, shows the amplitude and phase of the proposed 32$\times$32 structure of LCM-RIM. The reflection characteristics of the proposed design remain stable. For a large-scale array, the mutual coupling resulting from the close spacing between the elements is increased, which can lead to significant variations in the amplitude and phase responses. However, the proposed design has been meticulously optimized to address these issues. The unit cell geometry and layout have undergone full-wave simulation for thorough refinement. Furthermore, using a low-loss substrate (such as ROGERS 4003C) and maintaining consistent periodicity across the array ensures stable phase and amplitude responses throughout the 32$\times$32 structure, although the design has a slight frequency shift. As shown in Fig. 9a, and b , when the operating frequency deviates from the design value, the reflection phase response of the unit cell also shifts. For example, a nominal $180^\circ$ phase shift may reduce to $179^\circ$, depending on the direction and magnitude of the frequency offset. Such phase deviations can negatively impact beamforming performance and system efficiency. A phase tolerance of $\pm 30^\circ$ is commonly reported in the literature as acceptable for practical implementations ^64^, with negligible effects on beamforming accuracy and performance. To address this, adaptive tuning can be implemented using the proposed AlGaAs PIN diodes. In the current LCM-RIM design, we aim to maintain phase stability as the array scales to 32$\times$32 elements. Enhancing robustness to frequency variation will be a focus of future design optimization and experimental validation. Analytical array factor analysis and system-level simulations confirm that such minor phase offsets result in negligible beam steering error ($<0.1^\circ$) and $<0.5$ dB gain reduction, preserving the directional performance. Table 2 lists a design specification for the proposed 32 LCM-RIM in detail.

The proposed LCM-RIM utilizes AlGaAs PIN diodes with a forward bias voltage of $1.33\,\text {V}$ to achieve 1-bit phase control. Each diode consumes between 3 and $5\,\text {mA}$ of current when forward-biased, resulting in a power consumption of approximately 4 to $6.8\,\text {mW}$ per element. For a $32 \times 32$ RIM array with 1024 elements, the total worst-case diode biasing power ranges from $4.09\,\text {W}$ to $6.81\,\text {W}$. However, with practical addressing schemes such as row-column or time-multiplexed control, only a subset of diodes are actively biased at any moment, reducing the average diode power consumption to approximately 2–$3.5\,\text {W}$. In addition to the diode biasing, the control circuitry, including shift registers, a microcontroller (e.g., Arduino Mega), level shifters, and voltage regulators, contributes an estimated 1.1–$1.6\,\text {W}$ to the total system power. This brings the overall power consumption of the LCM-RIM system to approximately 3.1–$5.1\,\text {W}$ under typical operating conditions, and up to 5.2–$8.4\,\text {W}$ under worst-case continuous biasing. Despite these additional overheads, the system maintains a substantially lower power profile compared to active beamforming solutions that require high-power RF chains.

In terms of physical deployment, the 32$\times$32 LCM-RIM array measures 384 mm $\times$ 320 mm, with each unit cell sized at 12 mm $\times$ 10 mm. The compact, planar structure is well-suited for installation on walls or other fixed surfaces, making it ideal for enhancing signal strength in larger indoor environments. Additionally, the modular design allows for easy scalability to larger arrays (e.g., 64$\times$64 or 128$\times$128) by increasing the number of unit cells. While scaling introduces challenges, such as increased coordination requirements for AlGaAs PIN diode control and more complex power distribution across the surface, the binary 1-bit configuration maintains a low-overhead control architecture.

**Table 2:** Specifications of the proposed 32$\times$32 LCM-RIM structure.

Specification	Value
Structure horizontal size	384 mm
Structure vertical size	320 mm
Spacing between one element form horizontally	0.5$\lambda$
Spacing between one element form vertically	0.4$\lambda$
Total namer of diodes	1024 PIN diodes
Total namer of array	32x32 (1024 )
Phase	$180^o$ (0 or $\pi$)
Center frequency	24.12 GHz

**Fig. 10:**
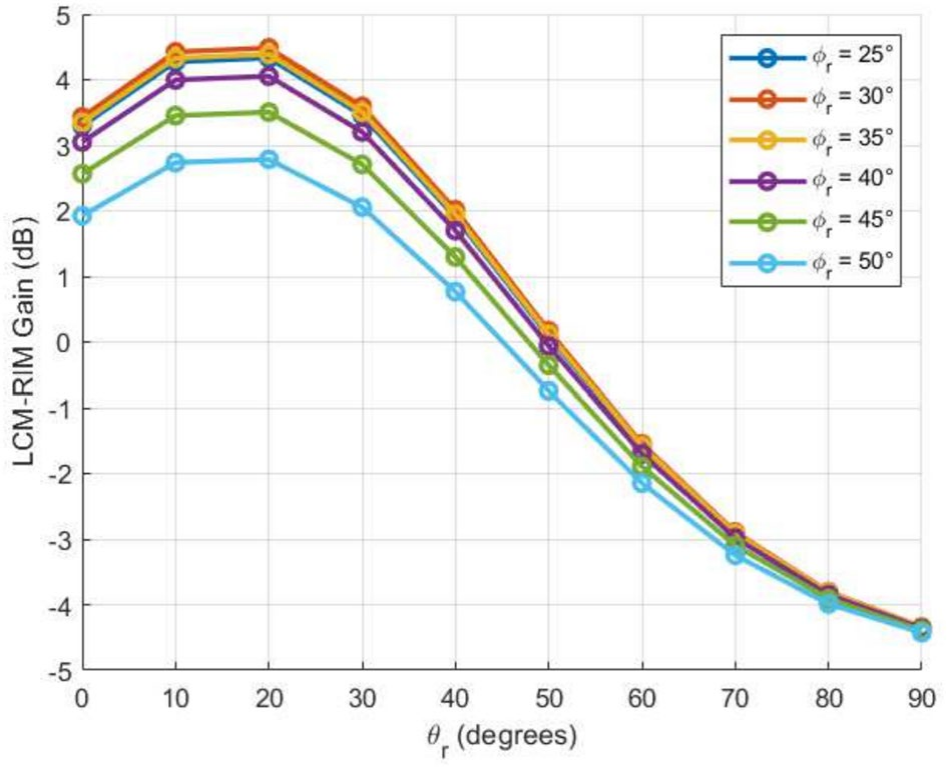
Characterization of the LCM-RIM’s reflected gain response over a range of reflection angles.

One of the most distinctive features of the proposed LCM-RIM is its directional array gain profile, which follows a Gaussian-like distribution concerning the receiver angle $\theta _r$. As illustrated in Fig. 10, the 32$\times$32 LCM-RIM array demonstrates a peak gain of up to 4.5 dB, with a symmetric roll-off as the receiver angle deviates from the optimal reflection direction. This smooth and predictable gain pattern is crucial for constructing an accurate analytical path loss model, as it enables consistent beamforming performance across various incident angles $\theta _i$. Compared to other RIM designs that have irregular or rapidly changing gain profiles, the Gaussian shape of the LCM-RIM gain facilitates simplified modeling through mathematical functions. This unique characteristic directly leads to improved received power and more reliable signal coverage in mmWave indoor communication environments.

In the next section, after presenting the proposed design, the role of this design in mitigating path loss in indoor environments is discussed.

## Path loss modeling for proposed LCM-RIM

Following the presentation of the M-shaped simulation structure in the previous section, this section introduces the theoretical path loss model, which aims to demonstrate how the proposed LCM-RIM can enhance wireless performance in indoor environments.

### System description of LCM-RIM-assisted indoor application

**Fig. 11:**
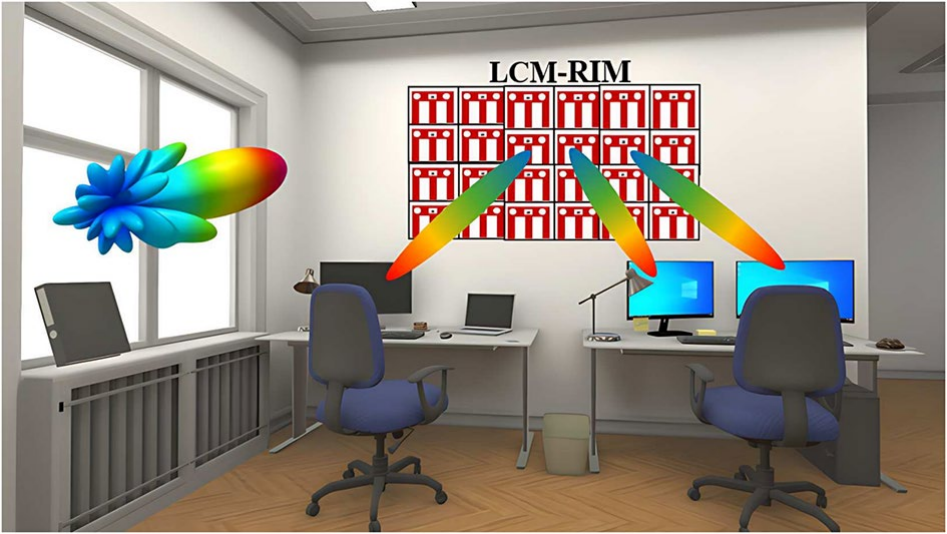
System description of LCM-LCM-RIM-assisted indoor application.

Figure 11 shows, that the proposed LCM-RIM can reflect the signal from the Base Station (BS), adjusting the suitable reflection amplitude and phase (0 or $\pi$) to the desired user. This design features an LCM-RIM installed on an indoor wall to enhance wireless coverage and performance. Each meta-surface element can dynamically adjust how it reflects or scatters incoming signals and focused beams that target specific user locations in the room. By considering these configurable elements, the system optimizes signal strength and reduces interference between users.

**Fig. 12:**
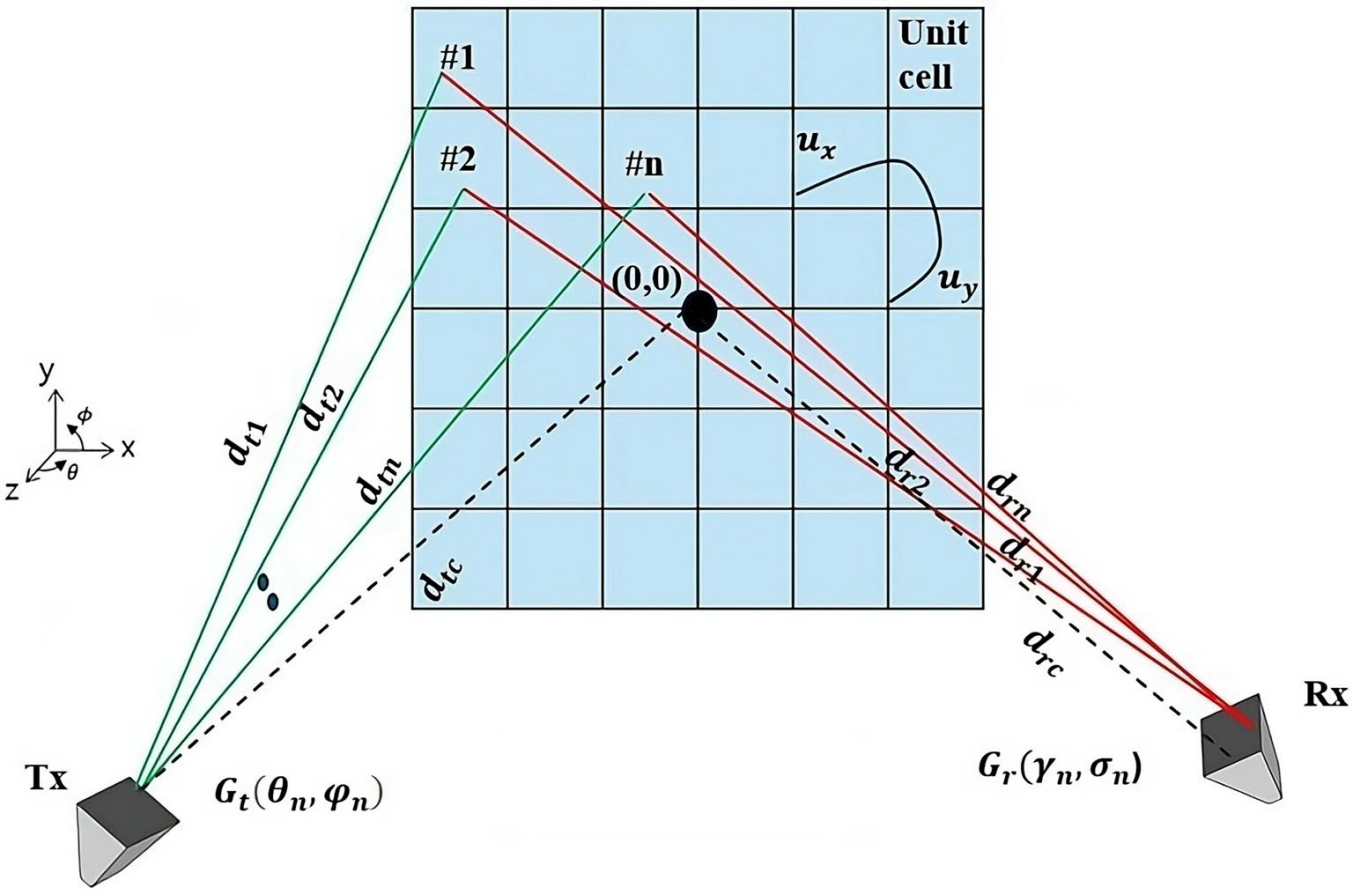
Scattering concept of LCM-RIM for path loss.

Figure 12 represents the concept of LCM-RIM in terms of its propagation paths. The EM waves emitted by the transmitter ($T_x$) can be reflected by a single unit cell of this RIM. The measurement of the received power can be calculated at the receiver ($R_x$), which can be assisted due to the propagation paths scenarios ( from $T_x$ to RIM and RIM to $R_x$); each of these paths can be expressed in the unit cell as^65^

6 $$\begin{aligned} P_r = P_t \left[ \sum _{n=1}^N G_{\text {t}} (\theta _n, \phi _n) G_{\text {unit}} (\alpha , \beta ) \left( \frac{\lambda }{4\pi d_n} \right) ^2 G_{\text {r}} (\gamma _n, \delta _n) G_{\text {unit}} (\alpha , \beta ) |\Gamma _n|^2 \left( \frac{\lambda }{4\pi d_n} \right) ^2 \right] . \end{aligned}$$

where $N$ is the total number of unit cells, $P_r$ is the received power, $P_t$ is the transmitted power, $G_{\text {t}}(\theta _n, \phi _n)$ and $G_{\text {r}}(\gamma _n, \delta _n)$ are the gains of the $T_x$ and $R_x$ antennas, $G_{\text {unit}}(\alpha , \beta )$ is the gain of the unit cell, $\lambda$ is the wavelength, $d_n$ is the distance for the $n ^{th}$ unit cell, and $|\Gamma _n|$ is the reflection coefficient.

The following subsections consider the function of the LCM-RIM in mitigating path loss by analyzing factors such as the RIM unit cell gain patterns and effective received power. In the next subsection, we discuss the primary function of the LCM-RIM, which will be substituted into Eq. (6).

### Gain profile of the LCM-RIM unit cell

In this subsection, we enhance the received power model introduced in Eq. (6) by incorporating the Gaussian gain performance of the LCM-RIM. The effective aperture from antenna theory is used to calculate the gain of the proposed LCM-RIM’s single unit cell, which is represented as a Gaussian distribution. This Gaussian distribution is obtained by fitting the gain of the single unit cell at different incident angle values.

7 $$\begin{aligned} \text {(dB)} \quad G_{\text {unit}}(\theta _{\text {in}}, \phi _{\text {in}})&= A \exp \left( -\frac{(\theta _{\text {in}} - \theta _0)^2}{2\sigma ^2} - \frac{(\phi _{\text {in}} - \phi _0)^2}{2\sigma ^2}\right) + B A_{e}(\alpha , \beta ), \end{aligned}$$

8 $$\begin{aligned} \text {(dB)} \quad G_{\text {unit}}(\theta _{\text {ref}}, \phi _{\text {ref}})&= A \exp \left( -\frac{(\theta _{\text {ref}} - \theta _0)^2}{2\sigma ^2} - \frac{(\phi _{\text {ref}} - \phi _0)^2}{2\sigma ^2}\right) + B A_{e}(\alpha , \beta ) \end{aligned}$$

where, the constants are A = 9.409, $\theta _0$ = 15.598, $\phi _0$ = 30.579, B = – 34.929, $\theta _{\textbf {in}}$ represents the incident of the elevation angle, $\phi _{\textbf {in}}$ is the incident of the azimuth angle, $\theta _{\textbf {ref}}$ is the reflection of the elevation angle, $\phi _{\textbf {ref}}$ is the reflection of the azimuth angle and $A_{e}(\alpha ,\beta )$ is the effective aperture of the unit cell.

To accurately evaluate the performance of LCM-RIM, it is necessary to analyze the interaction of the indecent angles on the unit cell. The aperture is used to determine this gain, as it is the fraction of available power at the aperture compared to the flux density of incident power. This relation will allow us to replace it with the gain properties present in Eqs. (7) and (8). The aperture is known as the ratio of available power at the aperture, and the power flux density for the incident wave on the unit cell is related as

9 $$\begin{aligned} A_e = \frac{P_{\text {av}}}{S_{\text {inc}}} \end{aligned}$$

where $\ P_{\text {av}}$ represents the available power and $S_{\text {inc}}$ denotes the power flux density of the incident wave.

Since the unit cell has the main EM fields radiated by the $T_x$ antenna, the majority of the incident power was located on the surface of the unit cell, which the effective aperture of the LCM-RIM unit cell can represent as the projected aperture area in the direction of the $T_x$ antenna.

**Fig. 13:**
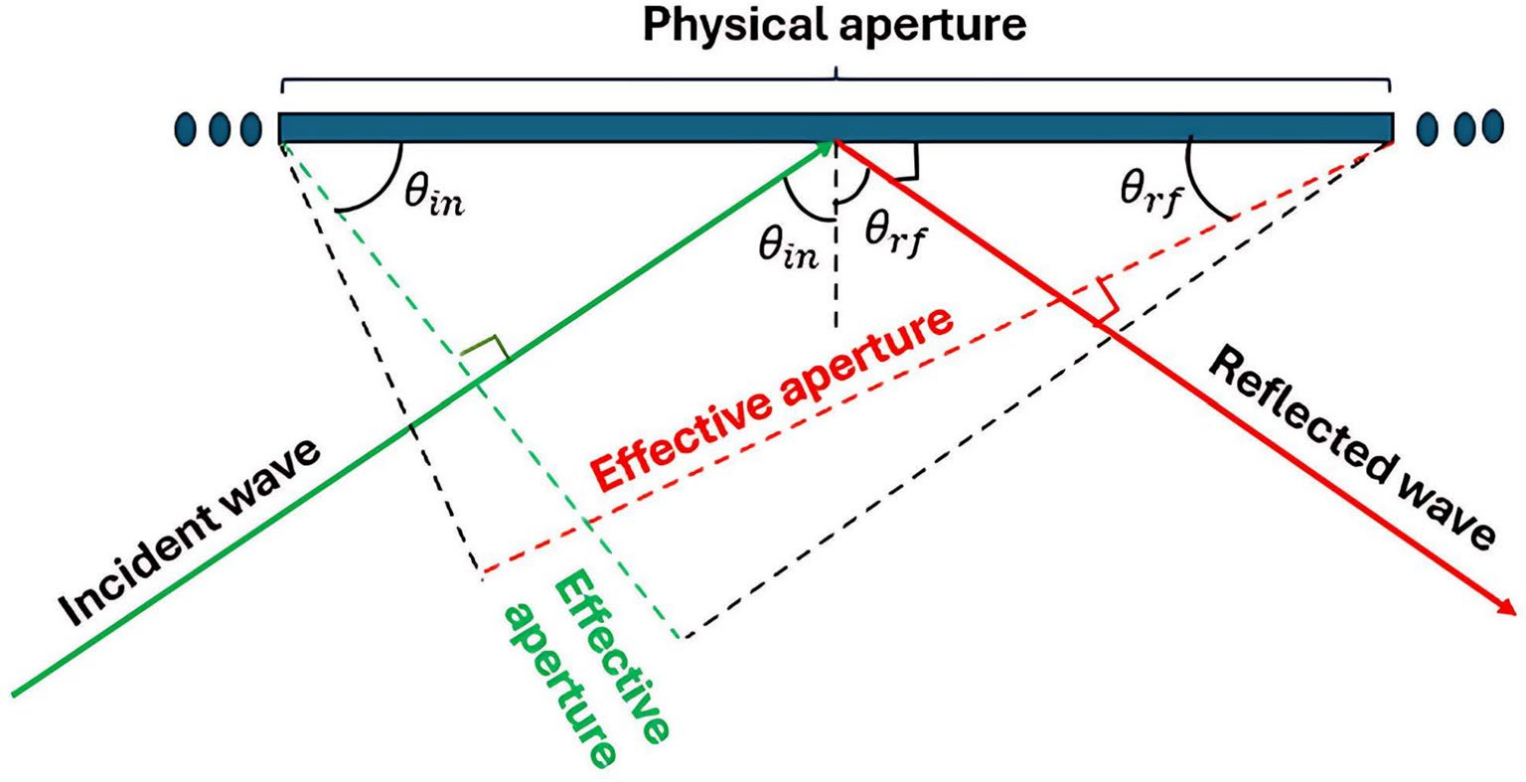
Effective aperture of LCM-RIM.

In Fig. 13, the effective aperture of the unit cell is graphically depicted when both $T_x$ and $R_x$ are found inside the azimuth plane ($\phi _{\text {in}}$) is zero, defining the concept of a 3D environment when LCM-RIM is in the projected aperture *x* and *y*, where the ($\theta _{\text {in}}$ and $\theta _{\text {rf}}$) is in the normal direction. Therefore, the aperture can determine the aperture efficiency as in Eqs. (10a) and (10b).

10a $$\begin{aligned} A_e (\alpha , \beta )&= A \exp \left( -\frac{(\theta _{\text {in}} - \theta _0)^2}{2\sigma ^2} - \frac{(\phi _{\text {in}} - \phi _0)^2}{2\sigma ^2}\right) + B A_{ph}(\alpha , \beta ) \cos (\theta _{\text {in}}), \end{aligned}$$

10b $$\begin{aligned} A_e (\alpha , \beta )&= A \exp \left( -\frac{(\theta _{\text {ref}} - \theta _0)^2}{2\sigma ^2} - \frac{(\phi _{\text {ref}} - \phi _0)^2}{2\sigma ^2}\right) + B A_{ph}(\alpha , \beta ) \cos (\theta _{\text {rf}}). \end{aligned}$$

where $A_{ph}(\alpha , \beta )$ is the physical aperture area of the unit cell.

In practice, the effective aperture area of the unit cell varies because it depends on the $\theta _{in}$ and $\theta _{\text {rf}}$, which differ depending on the position of the cells. Assuming that the aperture is a single value based on the center point of the LCM-RIM, due to the long distance between the LCM-RIM and the $T_x$, we can define the incident and reflected angles as $(\theta _{i})$ and $(\theta _{r})$ and we can rewrite Eq. (6) when substituting Eqs. (10a) and (10b) as

11 $$\begin{aligned} P_r&= P_t \left( \frac{A_{ph}}{4\pi }\right) ^2 \cos (\theta _{\text {i}}) \cos (\theta _{\text {r}}) \sum _{n=1}^N \Bigg [\frac{G_t (\theta _n, \phi _n)}{d_n^2} \times \Bigg ( A \exp \left( -\frac{(\theta _{\text {in}} - \theta _0)^2}{2\sigma ^2} - \frac{(\phi _{\text {in}} - \phi _0)^2}{2\sigma ^2}\right) \nonumber \\&\quad + B A_{ph}(\alpha , \beta ) \cos (\theta _{\text {in}}) \Bigg ) \Bigg ] \nonumber \\&\quad \times \sum _{n=1}^N \Bigg [\frac{G_r (\gamma _n, \delta _n) |\Gamma _n|^2}{d_n^2} \times \Bigg ( A \exp \left( -\frac{(\theta _{\text {ref}} - \theta _0)^2}{2\sigma ^2} - \frac{(\phi _{\text {ref}} - \phi _0)^2}{2\sigma ^2}\right) + B A_{ph}(\alpha , \beta ) \cos (\theta _{\text {ref}}) \Bigg ) \Bigg ] \end{aligned}$$

where $(\theta _{i})$ and $(\theta _{r})$ are the incident and reflected angles from the center of the LCM-RIM.

From Eq. (5), the $R_x$ has maximum power when the reflected angle aligns in the normal direction of LCM-RIM with a fixed incident angle and decreases according to the cosine of the incident angle. Simultaneously, the maximum power is received when the $T_x$ is positioned in the normal direction relative to the LCM-RIM surface. As the angle of incidence deviates from the normal, the $P_r$ decrease follows a cosine dependence.

### Beam efficiency of LCM-RIM

**Fig. 14:**
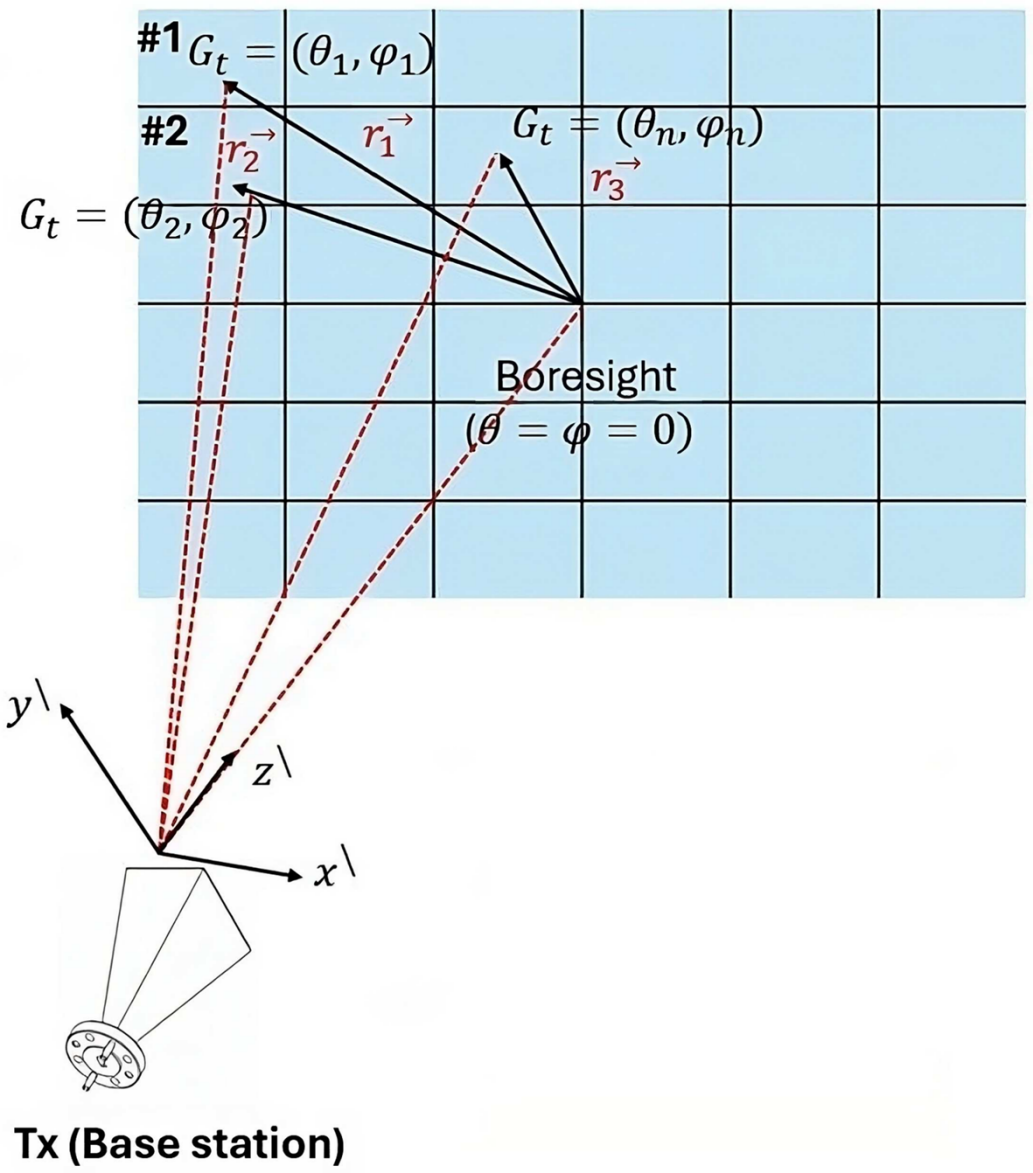
$P_r$ at LCM-RIM for a scenario of the small distance between the $T_x$ and LCM-RIM.

The LCM-RIM integrates seamlessly into various wireless communication systems, adapting to user requirements. When the distance between $T_x$ and LCM-RIM is too short or the LCM-RIM size is only marginally larger than necessary, the single-unit cell module introduces distinct variations in the reflection characteristics, affecting $P_r$, as illustrated in Fig. 14.

Traditional path loss models often assume uniform power reception across all unit cells of the RIM. To enhance the accuracy of our path loss model, we calculate the power of each unit cell. In this subsection, the power intensities incident on the unit cells from the $T_x$ antenna are accurately calculated for use in the path loss model. These values are derived based on the radiation pattern of the $T_x$ antenna and the specific configuration of the LCM-RIM-aided environment.

In theory, the gain of the antenna can be approximated as rectangular radiation, as in^66^. We derive the gain direction from the concept of the antenna radiation pattern, and can be written as

12 $$\begin{aligned} G&= \frac{4\pi }{\sin (\text {Bw}_{\theta _{\text {in}}}) \sin (\text {Bw}_{\phi _{\text {in}}})} , \end{aligned}$$

where, $\text {Bw}_{\theta _{\text {in}}}$ and $\text {Bw}_{\phi _{\text {in}}}$ represent the bandwidth along the axis, and *G* is the gain of the antenna. Since, $T_x$ has a high gain with a narrow beamwidth, we can form Eq. (12) and rewrite it as

13 $$\begin{aligned} G&= \frac{4\pi }{\text {Bw}_{\theta _{\text {in}}} \text {Bw}_{\phi _{\text {in}}}} , \end{aligned}$$

However, Eq. (13) represents the gain of the antenna in the ideal case, which radiates in a rectangular area. To solve this, we can rewrite it as^66^

14 $$\begin{aligned}&G=\varepsilon _m \frac{4\pi }{Bw_{\theta _{\text {in}}} Bw_{\phi _{\text {in}}} },\nonumber \\&\varepsilon _m = \frac{\Omega _m}{\Omega _a} \end{aligned}$$

where $\varepsilon _m$ is the beam efficiency, $\Omega _m$ is the solid angle of the beam, and $\Omega _a$ is the total pattern. The beam efficiency measures the propagation of power focused in the main beam to the total radiated power. In the ideal case, the efficiency equals 1.

We take Eq. (14) into account to consider the effect of beam efficiency on the power distribution of the unit cells. This equation will be used in the next subsection when substituted into the $P_r$ for the proposed model to refine our path loss calculations.

### Angle of the LCM-RIM unit cell

As discussed in section “Introduction”, the electric field of the incident wave, described in Eq. (2), can generate an induced current in the $x- direction$ direction. The LCM-RIM adjusts this current by tuning the surface impedance of each element according to Snell’s law.

When an LCM-RIM reflects a signal towards the angle $\theta _{rf}$, then the amplitude of the scattered field at any observation angle is $( \theta _s \in \left[ -\frac{\pi }{2}, \frac{\pi }{2} \right] )$. We can rewrite the $P_r$ for the proposed model by incorporating Eq. (14) as

15 $$\begin{aligned} P_r&= P_t \left( \frac{A_{ph}}{4\pi }\right) ^2 \cos (\theta _{\text {i}}) \cos (\theta _{\text {r}}) \sum _{n=1}^N \Bigg [\frac{G_t (\theta _n, \phi _n)}{d_n^2}\times \Bigg ( A \exp \left( -\frac{(\theta _{\text {in}} - \theta _0)^2}{2\sigma ^2} - \frac{(\phi _{\text {in}} - \phi _0)^2}{2\sigma ^2}\right) \nonumber \\&\quad + B A_{ph}(\alpha , \beta ) \cos (\theta _{\text {in}}) \Bigg ) \Bigg ] \nonumber \\&\quad \times \sum _{n=1}^N \Bigg [\frac{G_r (\gamma _n, \delta _n) |\Gamma _n|^2}{d_n^2} \times \Bigg ( A \exp \left( -\frac{(\theta _{\text {ref}} - \theta _0)^2}{2\sigma ^2} - \frac{(\phi _{\text {ref}} - \phi _0)^2}{2\sigma ^2}\right) + B A_{ph}(\alpha , \beta ) \cos (\theta _{\text {ref}}) \Bigg ) \Bigg ]\nonumber \\&\quad \times \left( \frac{\sin \left( \frac{\pi b}{\lambda } (\sin \theta _s - \sin \theta _{rf})\right) }{\frac{\pi b}{\lambda } (\sin \theta _s - \sin \theta _{rf})}\right) ^2 \end{aligned}$$

Assuming that ideal case where the $\theta _s$ = $\theta _{rf}$^67,68^, the $P_r$ can be simplified as

16 $$\begin{aligned} p_r = G_t Gr \left[ A exp \left( \frac{\theta _i-\theta _o}{2\sigma ^2}\right) ^2+B\right] cos^2(\theta _i), \end{aligned}$$

Therefore, the $P_r$ between the $T_x$ and $R_x$ through the $n^{th}$ element (assuming the only path loss problem) is

17 $$\begin{aligned} p_r = P_t N \frac{G_t Gr}{d_t^2 d_r^2} \left[ A exp \left( \frac{\theta _i-\theta _o}{2\sigma ^2}\right) ^2+B\right] cos^2(\theta _{i}) \sum _{n=1}^{N}\left| \Gamma _{n}^2 \right| \end{aligned}$$

where, $d_t$ and $d_r$ are the distances measured from the center of the LCM-RIM. This formulation significantly enhances the $P_r$ by mitigating the effects of path loss through intelligent beamforming. The presence of *N* LCM-RIM elements introduces an array gain that scales the $P_r$. Additionally, the $\cos ^2(\theta _i)$ factor ensures that power is maximized when the LCM-RIM is illuminated near its normal axis. Most importantly, the LCM-RIM dynamically adjusts the reflection coefficients $\Gamma _n$ to enforce constructive interference at the $R_x$, effectively overcoming the signal attenuation introduced by the propagation distances $d_t$ and $d_r.$

In the following section, we will evaluate the performance of the LCM-RIM path loss model across various scenarios, including its performance at different transmitter-receiver distances and angles of incidence.

## Simulation-based validation of LCM-RIM pathloss

In this section, the theoretical framework presented in the previous section is validated by simulating the setup using a MATLAB simulation. The focus is on an LCM-RIM deployed in a real indoor environment to mitigate path loss (assuming no other noise). In the setup, the BS operates as a single-$T_x$ antenna and a single $R_x$ antenna. Simulations are performed at an operating frequency of 24.12 GHz, with a fixed transmitter power of 1 W and antenna gains of 10.98 dB for both $T_x$ and $R_x$. The distance from $T_x$ to the proposed LCM-RIM is set at 38 m (assuming that the proposed LCM-RIM is on the wall as in Fig. 12).

**Fig. 15:**
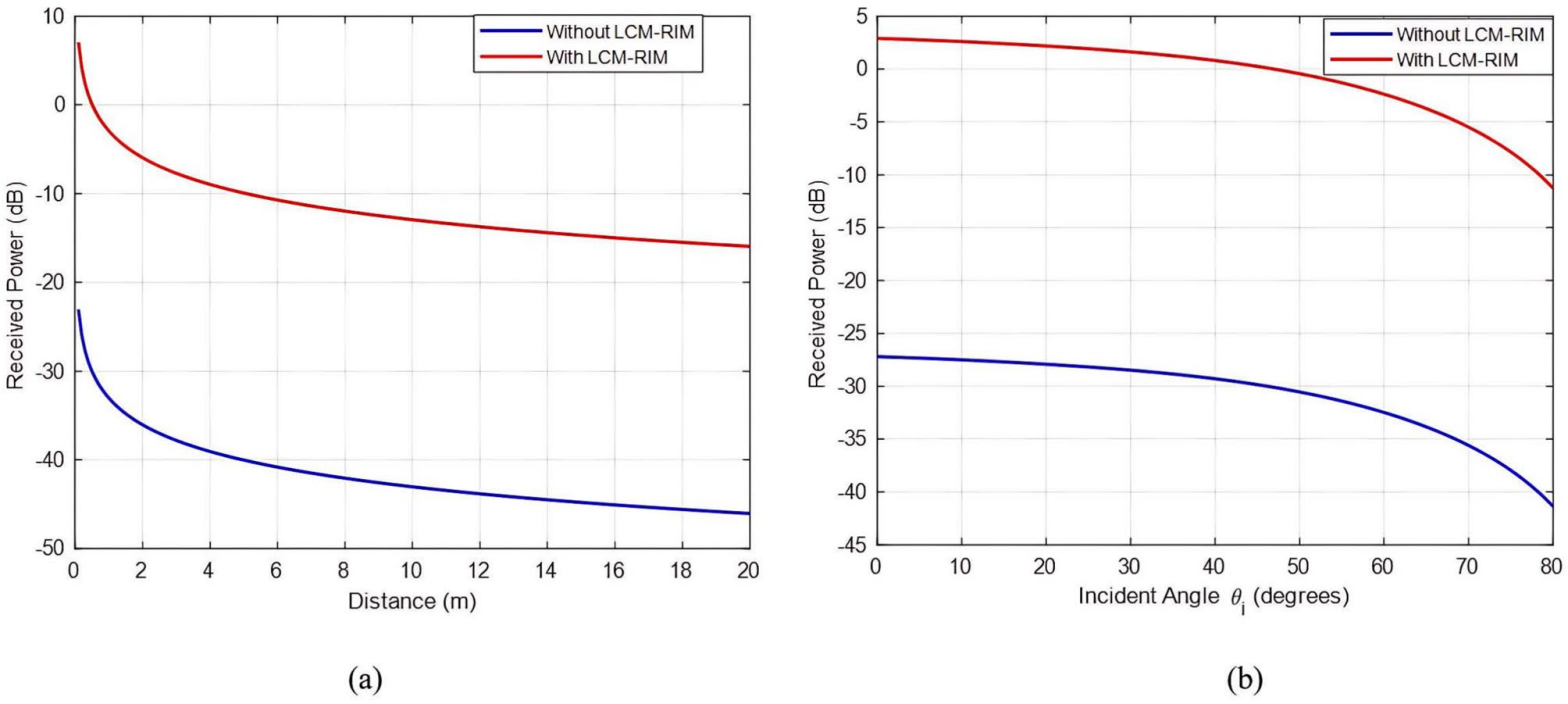
(**a**) Received power ($P_r$) vs. LCM-RIM-to-Rx deferent distance for $\theta _i = 30^\circ$ and $\phi _i = 0^\circ$, (**b**) Received power ($P_r$) vs. incident angle $\theta _i$ (0$\circ$ – 80$\circ$) at a fixed 5 m distance. The 32$\times$32 LCM-RIM yields up to 15 dB enhancement over the baseline scenario.

In the first configuration, the distance between the proposed LCM-RIM and the $R_x$ is varied from 0 m to 20 m, while the distance from the LCM-RIM to the $T_x$ remains fixed at 38 m. The $\theta _i$ is set to $30^\circ$, and $\phi _i$ is $0^\circ$. The LCM-RIM employs a 32$\times$32 element array to control phase shifts. Figure 15a shows the $P_r$, in (dB) at the $R_x$, calculated using the LCM-RIM path loss model, as a function of the LCM-RIM-to-$R_x$ separation. The results show a decrease in $P_r$ as $R_x$ moves further from the LCM-RIM, consistent with the free-space path loss trends; the maximum $P_r$ occurs when $R_x$ is closest to the LCM-RIM. Moreover, a comparison with a baseline scenario (without LCM-RIM) shows that the LCM-RIM can enhance $P_r$ by approximately 15 dB.

In the second configuration, the impact of varying the $\theta _i$ on the $P_r$ is analyzed, with the LCM-RIM-to-$R_x$ distance fixed at 5 m and the LCM-RIM maintained as a 32$\times$32 array. The angle $\theta _i$ is swept from $0^\circ$ to $80^\circ$, simulating deviations from the optimal alignment between the $T_x$ and the LCM-RIM. Figure 15b shows that the $P_r$ decreases significantly as it $\theta _i$ diverges from the near-normal incidence ($0^\circ$). This trend arises from the angular sensitivity of the LCM-RIM’s reflection gain, which follows a Gaussian-shaped profile modeled by a $\cos ^2(\theta _i)$ dependence in the path loss equation. At larger angles, the effective aperture of the LCM-RIM elements and their reflection efficiency diminish due to geometric projection losses and phase misalignment across the array.

**Fig. 16:**
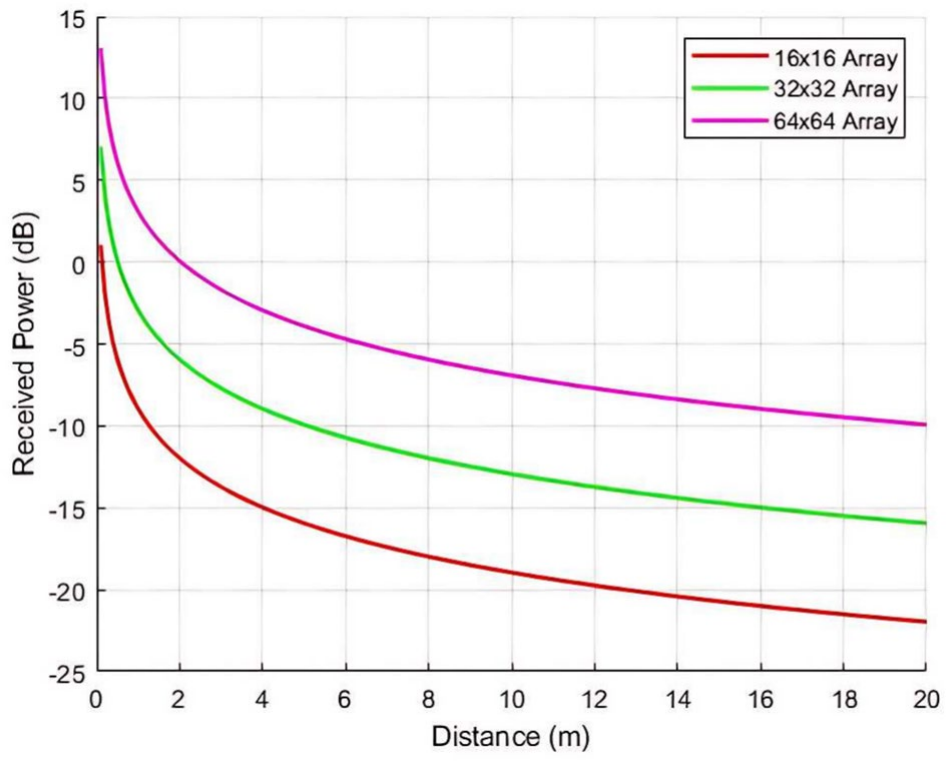
$P_r$ at various LCM-RIM array: 16x16, 32x32 and 64x64.

In the final configuration, the $\theta _i$ is maintained consistent with the previous setup. This configuration evaluates the impact of LCM-RIM array size on $P_r$ across varying $R_x$ distances. Three array configurations are compared: 16$\times$16, 32$\times$32, and 64$\times$64 elements. As illustrated in Fig. 16, the $P_r$ increases significantly with larger LCM-RIM arrays at all distances. The 64$\times$64 array achieves the highest $P_r$ due to its larger effective aperture, which enhances the LCM-RIM’s ability to focus and redirect incident EM waves toward the $R_x$. Conversely, the 16$\times$16 array exhibits the lowest power profile, with a pronounced decline in $P_r$ as distance increases.

These results confirm the theoretical model and illustrate how the proposed LCM-RIM can effectively improve indoor wireless coverage. In all test configurations, the observed trends are consistent with the predictions of the path loss model.

## Experimental setup and measurement procedures

After validating the numerical model of path loss of the proposed LCM-RIM, this section will present the measurement approach for physical implementations. This includes the setup for measurements and a beam steering technique to measure the $P_r$. Finally, we will discuss practical deployment challenges.

### System setup for measurements

**Fig. 17:**
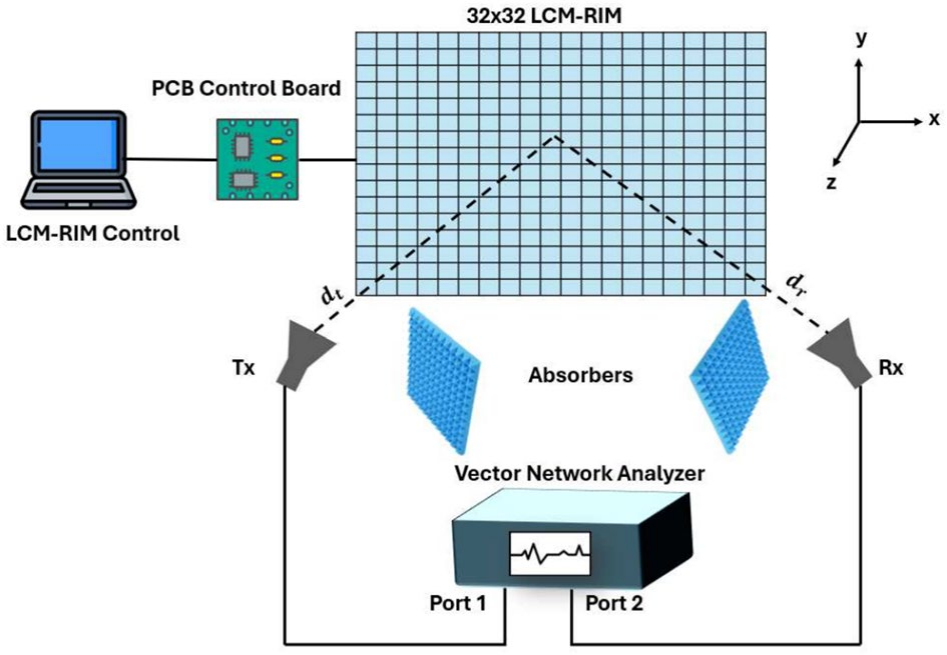
LCM-RIM measurement setup.

The measurement setup, as illustrated in Fig. 17, is implemented outside the coherent chamber to evaluate the real-world performance of the LCM-RIM in an indoor environment. This setup is essential in analyzing the performance of the LCM-RIM in high-frequency communication scenarios. The core component of the setup is a 32$\times$32 LCM-RIM, which is centrally placed to reflect the signal from the $T_x$ to the $R_x$. The LCM-RIM is connected to a control system that includes a computer and a PCB control board, allowing for dynamic adjustments of reflection coefficients. A Vector Network Analyzer (VNA) is utilized to measure the transmission parameter S between the Tx and Rx antennas, which measures the $S_{21}$ measure of path loss.

In the considered LCM-RIM-assisted indoor wireless system, beam steering is utilized as an essential technique to dynamically track the receiver as it moves through various angular positions. The LCM-RIM comprises an array of unit cells equipped with AlGaAs PIN diodes, allowing fast switching between ON and OFF states. These diodes are controlled via a control board, which updates the LCM-RIM configuration in real time based on the receiver’s location or AoA. As the $T_x$ remains fixed and the $R_x$ scans through various positions, the LCM-RIM adaptively alters its reflection pattern to maintain a high-gain communication link^69^.

### Beam steering technique

**Fig. 18:**
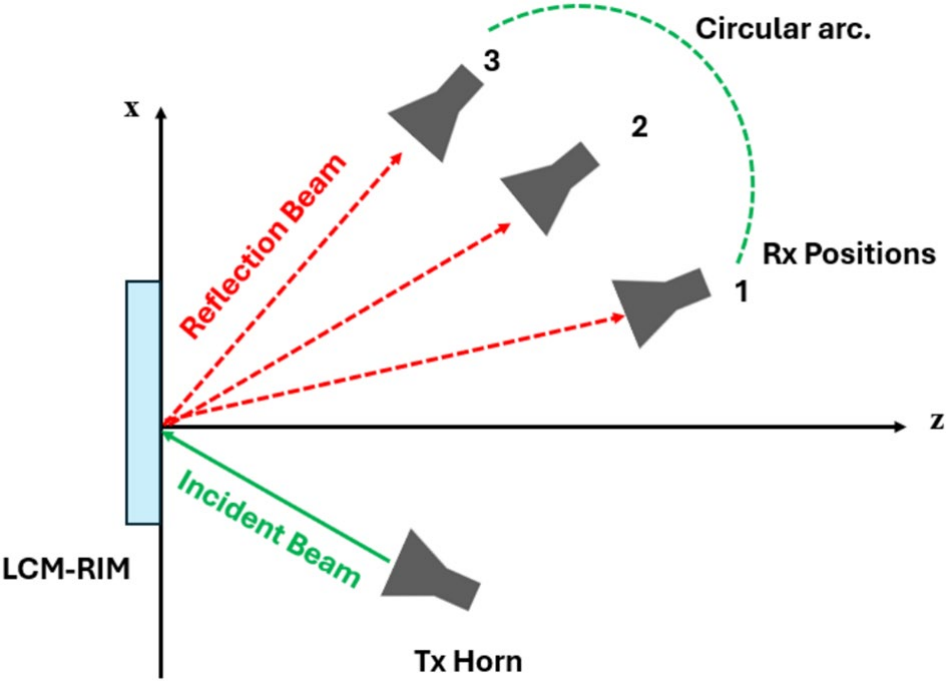
Schematic representation of LCM-RIM-assisted wave reflection, in which a $T_x$ incident signal is diverted to several $R_x$.

The beam steering technique is employed to measure the $P_r$ corresponding to different reflection angles. In Fig. 18, the $T_x$ antenna is fixed at a specific incident angle relative to the LCM-RIM, while the $R_x$ antenna is systematically repositioned to detect the reflected beam across a range of angles. For instance, at a given $T_x$ angle, the $R_x$ horn antenna is rotated from $10^\circ$ to $90^\circ$ in an anticlockwise direction. This is accomplished by physically moving the Rx antenna along a defined arc, allowing it to capture the reflected signal power at various angular positions. At each step, the reflected beam is observed, and the corresponding received power is measured using a VNA^64^.

To ensure robust performance in dynamic indoor environments, the proposed LCM-RIM framework incorporates a beam steering mechanism that enables real-time adaptation to changes such as user mobility and object displacement. By continuously estimating the angular position of the user, the LCM-RIM can steer its beam accordingly, allowing for precise tracking of the user’s location. The LCM-RIM then dynamically adjusts both the reflection amplitude and phase of each RIM element to optimize the signal quality at the receiver. This dynamic reconfiguration capability ensures that the system maintains high performance even in scenarios with varying user densities and environmental changes. In the next subsection, beam steering technique will be explained in detail.

### Practical implementation considerations of LCM-RIM for large-scale systems

The proposed LCM-RIM shows promising EM performance in full-wave simulations. However, transitioning to practical hardware deployment presents several engineering and system-level challenges. This section explores the feasibility of real-world implementation and highlights key considerations for scaling the RIM to large array sizes, such as 32$\times$32 elements. The proposed LCM-RIM shows promising EM performance in full-wave simulations. Transitioning to practical hardware deployment poses several engineering and system-level challenges. This section examines the feasibility of real-world implementation and highlights key considerations for scaling the RIM to large array sizes, such as 32$\times$32 elements. Table 3 summarizes main hardware and wireless-level challenges along with potential solutions.

**Table 3:** Practical considerations for implementing LCM-RIM in Large-Scale systems.

Aspect	Challenge	Potential solution
Control and Latency	Increased with large arrays	Use microcontroller with row-column addressing
Environmental Factors	extreme temperatures, and harmful substances	Conduct environmental testing
PCB Circuit Design	Bias line routing may interfere with RF paths	Careful bias decoupling and routing strategies
Wireless System Issues	multipath fading	Adaptive beamforming

Table 4 presents a comprehensive comparison of existing works in the literature based on key evaluation criteria, including design complexity (defined in terms of the number of structural layers and the types of materials used), operating frequency, path loss modeling approach, and target application. This comparison highlights the distinct advantages and positioning of the proposed LCM-RIM relative to current state-of-the-art metasurface solutions.

It presents the main design and performance metrics for RIS/IRS applications. The study in^65^ operates at dual frequencies using different array sizes (up to 100$\times$102), which employs a free-space EM analysis. The approach in^67^ utilizes metamaterial-based elements arranged in a large-scale IRS configuration, analyzed through a far-field model based on physical optics to increase communication performance. Additionally, a study in^68^, utilizes high-complexity, phase-reconfigurable unit cells, each incorporating two PIN diodes in a 24$\times$24 array, and adopts an improved model that considers both incident/reflected gain and phase errors.

This work introduces a novel design of an M-shaped reconfigurable meta-surface with low complexity and low cost, making it suitable for real-world applications such as wall-mounted environments. This approach utilizes the concept of the effective aperture of the proposed unit cell gain pattern, allowing high phase control to mitigate path loss in indoor environments.

**Table 4:** Comparison of studies that mitigate path loss in wireless systems.

Aspect	Work1^65^	Work2^67^	Work3^68^	Work4^70^	Work5^52^	Proposed work (LCM-RIM)
Frequency	10.5 GHz/4.25 GHz	N/A	29 GHz	4.8 GHz / 5.35 GHz	28 GHz	24.12 GHz
Design Complexity	High	Moderate	High	Moderate	High	Low
Unit Cell Type	Regular square shape	Meta-material	Phase-unit cell	Meta-material	N/A	Phase-M shape
No. of diodes/ unit cell	N/A	N/A	2 PIN unit cell	N/A	N/A	1 AlGaAs
Array size	100x105 / 50x35 / 8x32	N/A	24x24	8x8 / 16x16	40x40	16x16 / 32x32 / 64x64
Path Loss Model	Free-space model based on EM	Far-field model	Gain and phase error	Free-space considering RCS	Theoretical RIS tunnel model (RT, GO, UTD, t-distribution)	Effective aperture-based and Gaussian gain fit
Application	RIS for wireless systems	IRS for wireless systems	RIS for 5G,6G	RIS for RFID communications	RIS-assisted tunnel communication	RIM for indoor systems

## Conclusion

This paper proposed a novel M-shaped low-complexity reconfigurable intelligent meta-surface (LCM-RIM) designed to operate at 24.12 GHz to enhance mmWave indoor wireless communication systems. The LCM-RIM features a compact, single-layer unit cell constructed with a low-loss Rogers substrate and utilizes AlGaAs PIN diodes to enable discrete phase control with minimal power consumption and hardware complexity. Its scalability was demonstrated through array configurations of 16$\times$16, 32$\times$32, and 64$\times$64 elements, all with 0.5$\lambda$ spacing, supporting efficient wall-mounted deployment in indoor environments such as conference rooms and office spaces. A numerical path loss model was developed by characterizing the angular-dependent gain of the LCM-RIM using a Gaussian distribution. MATLAB-based simulations validated the proposed system, showing up to 15 dB improvement in received signal power, confirming the design’s effectiveness for 6G indoor applications. While the proposed LCM-RIM structure has demonstrated significant performance improvements in simulation, this study is currently limited to full-wave EM modeling and MATLAB-based path loss analysis. Real-world implementation introduces several practical considerations not captured in the simulation, such as fabrication tolerances, measurement uncertainties, control latency of the diodes, and power distribution across large arrays. In future work, we plan to incorporate realistic indoor conditions—such as multipath, noise, and common scatterers—to evaluate the system’s behavior in complex environments. This real-world validation will bridge the gap between theoretical modeling and practical deployment, ensuring that the LCM-RIM design is robust, scalable, and effective for 6G indoor wireless communication systems.

## Data Availability

The datasets used and/or analysed during the current study are available from the corresponding author on reasonable request.
